# Integrating network pharmacology with molecular docking to rationalize the ethnomedicinal use of *Alchornea laxiflora* (Benth.) Pax & K. Hoffm. for efficient treatment of depression

**DOI:** 10.3389/fphar.2024.1290398

**Published:** 2024-03-05

**Authors:** Nem Kumar Jain, Mukul Tailang, Balakumar Chandrasekaran, Nasha’t Khazaleh, Neelaveni Thangavel, Hafiz A. Makeen, Mohammed Albratty, Asim Najmi, Hassan Ahmad Alhazmi, Khalid Zoghebi, M. Alagusundaram, Hemant Kumar Jain

**Affiliations:** ^1^ School of Pharmacy, ITM University, Gwalior, Madhya Pradesh, India; ^2^ School of Studies in Pharmaceutical Sciences, Jiwaji University, Gwalior, Madhya Pradesh, India; ^3^ Faculty of Pharmacy, Philadelphia University, Amman, Jordan; ^4^ Department of Pharmaceutical Chemistry and Pharmacognosy, College of Pharmacy, Jazan University, Jazan, Saudi Arabia; ^5^ Pharmacy Practice Research Unit, Department of Clinical Pharmacy, College of Pharmacy, Jazan University, Jazan, Saudi Arabia; ^6^ Department of General Medicine, Government Medical College, Datia, Madhya Pradesh, India

**Keywords:** *Alchornea laxiflora*, depression, network pharmacology, molecular docking, *in silico*, ethnomedicine, African traditional medicine

## Abstract

**Background:** Alchornea laxiflora (Benth.) Pax & K. Hoffm. (A. laxiflora) has been indicated in traditional medicine to treat depression. However, scientific rationalization is still lacking. Hence, this study aimed to investigate the antidepressant potential of A. laxiflora using network pharmacology and molecular docking analysis.

**Materials and methods:** The active compounds and potential targets of A. laxiflora and depression-related targets were retrieved from public databases, such as PubMed, PubChem, DisGeNET, GeneCards, OMIM, SwissTargetprediction, BindingDB, STRING, and DAVID. Essential bioactive compounds, potential targets, and signaling pathways were predicted using *in silico* analysis, including BA-TAR, PPI, BA-TAR-PATH network construction, and GO and KEGG pathway enrichment analysis. Later on, with molecular docking analysis, the interaction of essential bioactive compounds of A. laxiflora and predicted core targets of depression were verified.

**Results:** The network pharmacology approach identified 15 active compounds, a total of 219 compound-related targets, and 14,574 depression-related targets with 200 intersecting targets between them. SRC, EGFR, PIK3R1, AKT1, and MAPK1 were the core targets, whereas 3-acetyloleanolic acid and 3-acetylursolic acid were the most active compounds of A. laxiflora with anti-depressant potential. GO functional enrichment analysis revealed 129 GO terms, including 82 biological processes, 14 cellular components, and 34 molecular function terms. KEGG pathway enrichment analysis yielded significantly enriched 108 signaling pathways. Out of them, PI3K-Akt and MAPK signaling pathways might have a key role in treating depression. Molecular docking analysis results exhibited that core targets of depression, such as SRC, EGFR, PIK3R1, AKT1, and MAPK1, bind stably with the analyzed bioactive compounds of A. laxiflora.

**Conclusion:** The present study elucidates the bioactive compounds, potential targets, and pertinent mechanism of action of *A. laxiflora* in treating depression*. A. laxiflora* might exert an antidepressant effect by regulating PI3K-Akt and MAPK signaling pathways. However, further investigations are required to validate.

## 1 Introduction

Network pharmacology has been widely utilized for understanding the underlying molecular mechanism of various traditional medicine systems, including traditional Chinese medicine and Indian Ayurvedic system, via systematic collecting, synthesizing, predicting, and analyzing the gathered information related to bioactive molecular targets and pathways (Chandran and Patwardhan, 2017; Jiashuo et al., 2022). Similarly, network pharmacology has garnered wider attention in exploring ethnobotanical knowledge to add newer therapeutic options to the existing medicines for simple to severe diseases (Jiashuo et al., 2022). Network pharmacology is an emerging technology, along with the rapidly developing bioinformatics approach to deal with complex systems of medicine, including the Indian Ayurvedic system and the traditional Chinese medicine in which the One drug, one Target concept of the Western medicine system does not fit (Guo et al., 2019; Shamsi et al., 2022).

Various researchers have reported the use of integrated network pharmacology and molecular docking analysis to investigate the mechanism of multiple traditional Chinese medicines (TCM), for example, the Xiaoyaosan formula (Gao et al., 2018), Huanglian Jiedu decoction (Li et al., 2022), and Huangqi Sijunzi decoction (Cui et al., 2021). Guo et al. (2019) implemented network pharmacology-based prediction tools to characterize the molecular mechanism of the Zuojin pill on hepatocellular carcinoma. The study found that the therapeutic benefits of the Zuojin pill against hepatocellular carcinoma might be attributed to its ability to regulate the EGFR/MAPK, PI3K/NF-κB, and CCND1 signaling pathways. In another investigation, Ye et al. (2021) utilized a combination of network pharmacology, molecular docking, and molecular dynamics simulation to provide a theoretical basis for the clinical application of Yinchen wuling powder for hyperlipidemia therapy. Yinchen Wuling powder’s anti-hyperlipidemic effect was attributed to its various components (quercetin, isorhamnetin, and taxifolin) that interact with multiple targets (AKT1, IL6, VEGFA, and PTGS2) of hyperlipidemia. Similarly, network pharmacology has been applied to various complex disease conditions, such as Alzheimer, asthma, atherosclerosis, diabetes mellitus, cancer, COVID-19, etc. (Batool et al., 2022; Zhang Z. et al., 2022; Wang et al., 2022; Alnusaire et al., 2023; Rampadarath et al., 2023; Shamsol Azman et al., 2023).

Depression is a psychiatric disorder that clinically presents as mood and cognition changes and loss of interest persisting for at least 2 weeks (Lin et al., 2020). In 2020, the World Health Organization (WHO) declared it the second most significant disease to consider due to the rise in global burden (Gbadamosi et al., 2022). In Africa, depression accounts for 9% of the population, affecting about 30 million people. Nigeria alone harbors 7 million depression patients (Esan and Esan, 2016). With the limited availability of adequate and economical medication, there is a rise in cases of treatment-resistant depression in Africa (Gbadamosi et al., 2022). Owing to the non-affordability of costly medicines by the African diaspora, the use of indigenous therapies for the management of depression is reported in several ethnomedicinal studies (Wubetu et al., 2018). However, a few medicinal plants have been evaluated extensively for their antidepressant mechanism of action (Maina et al., 2020). An array of studies has revealed that medicinal plants may hold the key to discovering lead chemicals for innovative therapeutics against several neurological disorders (Wal et al., 2022; Wal et al., 2023 A.; Wal et al., 2023 P.; Alrouji et al., 2023; Nelson et al., 2023). Hence, research in this area could help develop effective management of psychiatric disorders in Africa.

*Alchornea laxiflora* (Benth.) Pax & K. Hoffm. (*A. laxiflora*), belonging to the family Euphorbiaceae is an underexplored African traditional botanical, listed in Cameroonian traditional pharmacopeia for various health issues (Sandjo et al., 2011). It has different vernacular names depending on the cultural and ethnic diversity in Africa. Some common African names include Ewe Pepe, Pepe, Ewe lya, and Gbogbonse (Oladunmoye and Kehinde, 2011). *A. laxiflora* is endemic to tropical African countries such as Nigeria, Cameroon, South Africa, Tanzania, Uganda, etc. Various phytochemicals have been isolated and reported to possess different pharmacological activities. Detailed information on phytochemistry and pharmacology of *A. laxiflora* has been reported by Jain et al. (2022b). As per the literature search, a wide array of ethnomedicinal uses are reported for various parts of *A. laxiflora,* including infectious disease, gastrointestinal disorders, inflammatory conditions, and neurological disorders such as anxiety, depression, insomnia, and epilepsy, but only a few indications have been scientifically validated. The decoction from the leaves of *A. laxiflora* is traditionally used in the Bafia, Bazou, and Foumbot regions of East and West Cameroon to treat depression. However, there is no pharmacological study available to support this traditional use (Ngnameko et al., 2019). Therefore, the present study aimed to generate networks to provide a rational and molecular mechanism of the ethnomedicinal use of *A. laxiflora* as an antidepressant botanical by bringing the bioactive compounds, molecular targets, and interacting pathways together.

## 2 Materials and methods

### 2.1 Methodological approach

**FIGURE 1:**
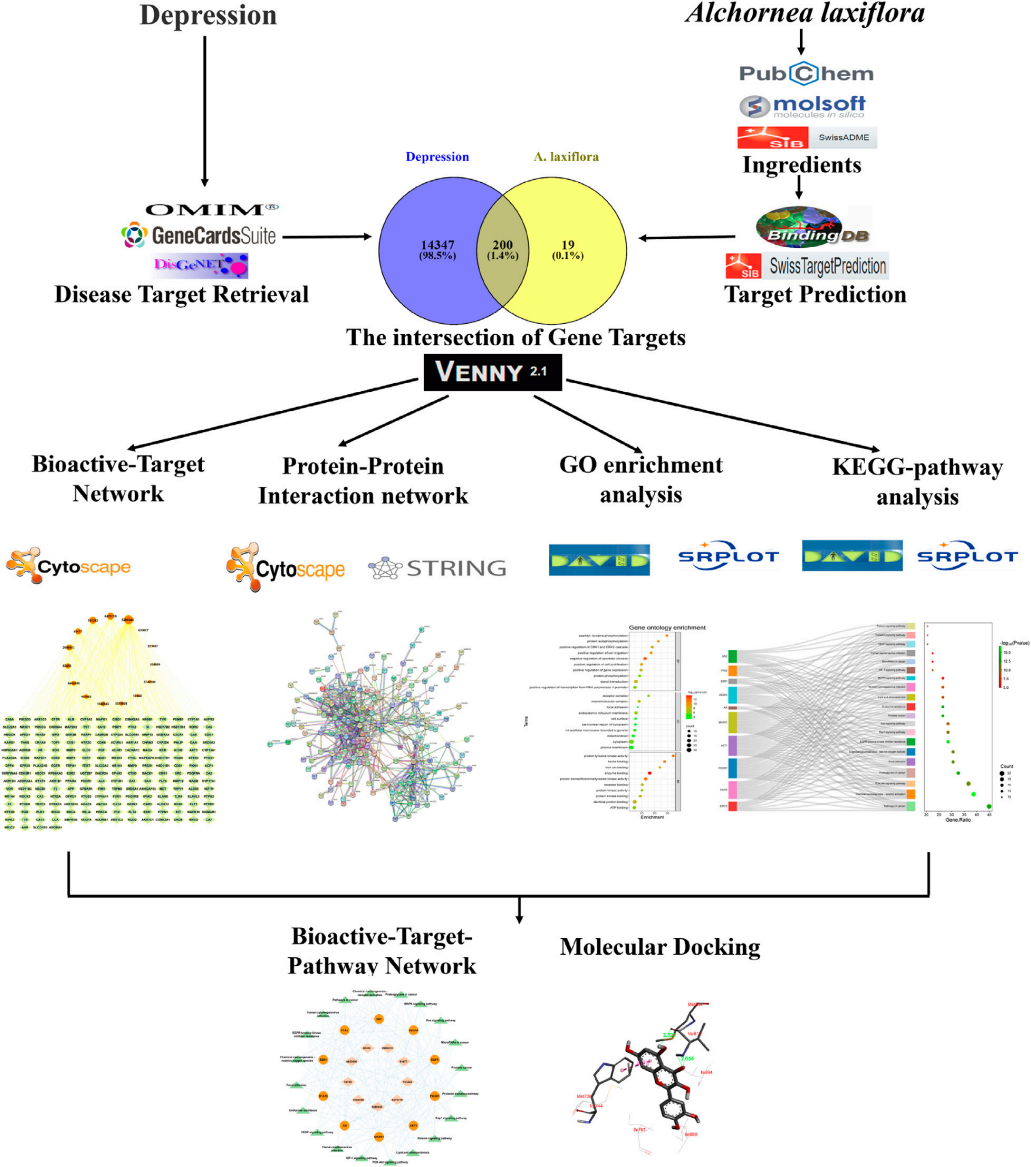
An outline of the methodological approach to elucidate the anti-depressant potential of *A. laxiflora*.

Various computational tools and databases were used to investigate and predict the bioactive compounds, potential gene targets, and pathways involved in the pharmacological network of *A. laxiflora* on depressive disorder. In addition, Molecular docking techniques were used to validate the potential underlying mechanisms (Jain et al., 2022a; Abunuwar et al., 2023; Najmi et al., 2023). An outline of the methodological approach in our current study is demonstrated in Figure 1.

### 2.2 Screening of bioactive compounds from *A. laxiflora*

The information on the phytoconstituents of *A. laxiflora* was retrieved from our previously published systemic review on the phytochemistry and pharmacology of *A. laxiflora* (Jain et al., 2022b). PubChem (https://pubchem.ncbi.nlm.nih.gov/), assessed on 10 February 2023, was used to collect each active ingredient’s molecular weight, chemical structures, canonical SMILES, and 2D SDfiles (Cheng et al., 2014). Components without Pubchem ID were discarded and were not included for further investigation (Xiao et al., 2022).

### 2.3 ADME and drug-likeliness screening

Later, all active ingredients screened from the literature survey were virtually analyzed for ADME characteristics using SwissADME (http://www.swissadme.ch/index.php) and Molsoft (https://molsoft.com/mprop/) online tools for oral bioavailability (OB) and drug-likeness (DL) scores, respectively, assessed on 16 February 2023, by entering PubChem ID of the screened bioactive compounds (James et al., 2015; Daina et al., 2017). Blood-brain barrier (BBB) score obtained from Molsoft was also used as screening criteria, cutoff value was set as >1 (Gupta et al., 2019). Compounds with OB ≥ 30% and DL ≥ 0.18 were regarded to have high OB and druggability, respectively, and were set as screening criteria (Shi et al., 2021; Wang et al., 2022). Three compounds with DL < 0.18 were added to the study based on extensive pharmaceutical activities. Using Lipinski’s rule of five, the ADME criteria were also applied, and compounds with >3 violations were excluded from the investigation (Lin et al., 2020).

### 2.4 Target genes associated with screened bioactive compounds and depression

Target genes of filtered bioactive compounds were predicted using SwissTargetprediction and BindingDB databases. SwissTargetprediction database (http://www.swisstargetprediction.ch/) predicts the most probable gene targets of the entered compound with the “*Homo sapiens*” setting. The database was searched with canonical SMILES of selected bioactive compounds in the SwissADME tool. The result was imported into the SwissTargetprediction database for the probable gene targets of *A. laxiflora* active ingredients (Gfeller et al., 2014). The predicted gene targets were further screened for reliability with a probability score ≥0.70.

The BindingDB (https://www.bindingdb.org/rwd/bind/index.jsp) database was queried with 2D SDFiles of selected active compounds to get predicted gene targets with high similarity (≥0.70). UniProt IDs from bindingDB results were used to gather official protein names, gene IDs, and organisms from the UniProtKB database (https://www.uniprot.org/). Predicted gene target data was compiled in an Excel file and limited target selection to “*H. sapiens*” as species (Gilson et al., 2016). Predicted targets of *A. laxiflora* bioactive compounds from both databases were combined and checked for duplicate data and their removal. Common targets were saved for further analysis.

Similarly, depression-related gene targets were retrieved using the keywords “depression, major depressive disorder, and mental depression” from the OMIM (https://www.omim.org/), GeneCards (https://www.genecards.org/) and DisGeNET (https://www.disgenet.org/home/) public disease databases (Amberger et al., 2015; Stelzer et al., 2016; Piñero et al., 2017). All the results were aggregated in a single file, and overlapping gene targets were used for further analysis. To identify the intersected depression-related gene targets intervened by *A. laxiflora* active ingredients, Venny 2.1.0 (https://bioinfogp.cnb.csic.es/tools/venny/) web tool was used (Wang Y. et al., 2021). Intersection gene targets of selected *A. laxiflora* bioactive compounds and depression were retrieved for subsequent network analysis and molecular docking validation.

### 2.5 Botanical-bioactive-target (BA-TAR) network construction

A bioactive-target (BA-TAR) network was constructed using Cytoscape v3.9.1 (https://cytoscape.org/) to investigate the multi-component relationship between bioactive compounds and predicted overlapping targets (Shannon et al., 2003). The bioactive compounds and intersecting gene targets were imported into Cytoscape v3.9.1 to construct and analyze the network’s topological structures using the cytoNCA plug-in with the “Degree value” setting. Compounds with high degree values were considered essential bioactive compounds for treating depression.

### 2.6 Protein-protein interaction (PPI) network construction

The overlapping targets of depression and *A. laxiflora* bioactive compounds were queried on a STRING v11.5 (https://string-db.org/) online database to identify possible inter-target interactions (Szklarczyk et al., 2019). The official gene names of common targets were searched in multiple proteins by official gene names and the “*H. sapiens*” setting. A high confidence score of 0.70 was selected as the minimum required interaction to increase the reliability of generated data (Alnusaire et al., 2023). The resulting PPI network was first exported in TSV and PNG format and later imported into Cytoscape v3.9.1 to build a visual PPI network and further topological analysis. The network analyzer in Cytoscape v3.9.1 was used to gather node and edge information (Ibrahim and El-Banna, 2021). The CytoNCA plug-in was used to select core targets based on degree centrality, betweenness centrality, and closeness centrality (Li et al., 2021). Network nodes with degree values two times the median values were chosen as core targets, and a sub-structure was drawn. The top 10 hub targets were also selected using the CytoHubba plug-in using the degree technique out of the 12 topological parameters.

### 2.7 Gene ontology (GO) and pathway enrichment analysis

To understand the mechanism of *A. laxiflora* bioactive compounds for the treatment of depression, the above potential antidepressant core targets were queried in the DAVID 2021 functional annotation tool (https://david.ncifcrf.gov/tools.jsp) for Gene ontology analysis and KEGG pathway enrichment analysis (Kanehisa and Goto, 2000; Jiao et al., 2012). The official gene symbols of the core targets were entered with *H. sapiens* as the selected species. Analyses yielded the top 10 GO biological processes (BP), molecular functions (MF), cellular component (CC) terms, and the top 20 KEGG pathways. We sorted the desired data by applying filters on gene count from largest to smallest, *p*-value <0.01, and FDR <0.01 (He et al., 2023). The GO terms, KEGG pathways bar plots, and bubble plots were prepared with SRPlot (https://www.bioinformatics.com.cn/en).

### 2.8 Bioactive-target-pathway (BA-TAR-PATH) network construction

Based on the PPI network and KEGG analysis, Cytoscape v3.9.1 was utilized to construct a BA-TAR-PATH network of *A. laxiflora* key bioactive compounds, hub gene targets, and the top 20 KEGG pathways. In a constructed network, nodes represent bioactive compounds, targets, and pathways, and edges indicate the interactions among the three.

### 2.9 Molecular docking simulations

The main bioactive compounds of *A. laxiflora* from the bioactive-target network and the core protein targets from the protein-protein interaction (PPI) implicated in the bioactive-targets-pathways network were docked using molecular docking. Further, this molecular docking assists in validating the binding affinity of bioactive phytoligands of *A. laxiflora*, leading to the prediction of their antidepressant activity.

The protein structures in the 3D format of the top six hub genes resulting from network pharmacology were retrieved from the RCSB PDB database (Berman et al., 2000). The selection criteria of the target protein 3D structures are as follows: (a) X-ray solved crystal structures with a better resolution or up to 2.5 Å were included; (b) if two or more numbers of 3D structures are available in the database, then, the better resolution is considered for the selection; (c) target structures bearing the bound ligands are selected with high priority; (d) the proteins isolated from human as an organism are preferably selected.

The core phytoligand’s two-dimensional (2D) structures were acquired from the PubChem database (https://pubchem.ncbi.nlm.nih.gov/), converted to three-dimensional (3D) structures using Chem3D 20.0 version software, and saved in PDB format. PyMOL 2.4.0 software was used to remove water and other non-protein molecules, separate the cognitive bound ligand from the binding site of the protein, repair missing atoms on residues, and add polar hydrogens (Mooers, 2020). Finally, AutoDock 4.2.1. and AutoDock Vina 1.1.2 software was used for the molecular docking tasks (Morris et al., 2009; Trott and Olson, 2010). The grid box for docking was built for each target protein’s binding sites and saved in. pdbqt format. The information on protein targets with their PDB IDs and center coordinates (x,y,z-centre) are mentioned in Table 1. The docking results were visualized and analyzed using BIOVIA Discovery Studio Visualizer 2021 (He et al., 2021).

**TABLE 1:** Information on protein targets and their center coordinates.

Target protein	PDB ID	Centre coordinates (x, y, z centre)
MAPK1	1TVO	6.43, −4.37, 16.44
SRC	2BDF	6.86, 39.09, 19.45
EGFR	2RGP	16.29, 34.87, 92.04
AKT1	3O96	8.37, −6.83, 12.62
STAT3	6NUQ	13.13, 55.61, 0.11
PI3KR1	6PYR	38.21, 13.22, 33.47

## 3 Results

### 3.1 Acquisition of *A. laxiflora* bioactive compounds

**FIGURE 2:**
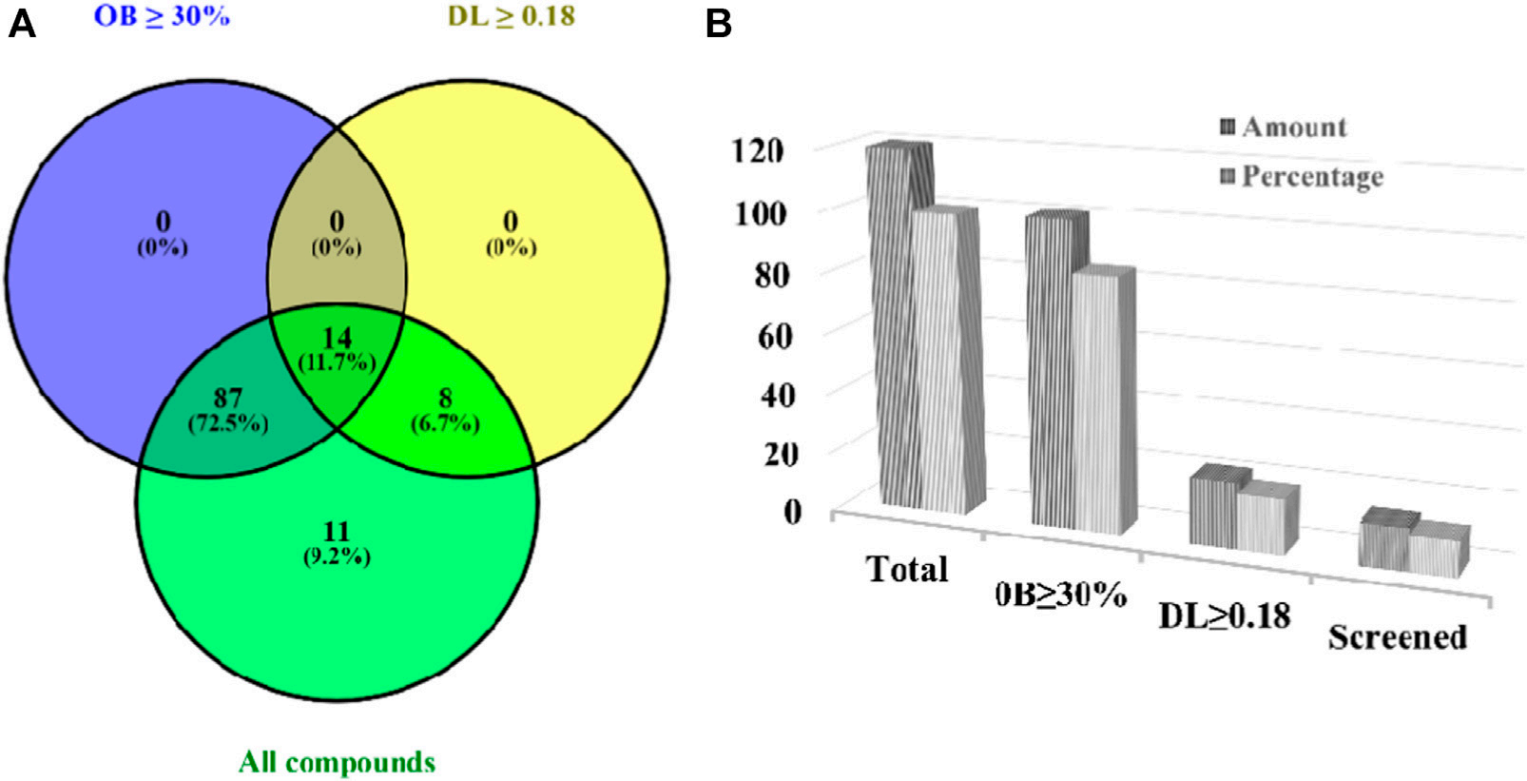
Screened bioactive compounds of *A. laxiflora* using two ADME-related properties; oral availability (OB) and drug-likeness (DL). Venn diagram **(A)** And Bar chart **(B)** Showing the number and percentage distribution of the bioactive compounds matching the screening criteria.

In our previously published systemic review on *A. laxiflora*, a total of 132 compounds were reported, comprising 43 fatty acids, 22 terpenoids, 19 phenolics, 13 flavonoids, 6 alkaloids, and other secondary metabolites (Jain et al., 2022b). Of the retrieved components, 12 compounds had no PubChem IDs and were excluded from the study. The 120 compounds of *A. laxiflora* were further studied for ADME and drug-likeness analysis using SwissADME and Molsoft online tools. Of 120 bioactive compounds, 14 met OB ≥ 30% and DL ≥ 0.18 screening criteria (Figure 2). Three more compounds such as capsaicin, dihydrocapsaicin, and 3-O-Methylellagic acid-3′-O-α-rhamnopyranoside with DL < 0.18 were also included in the database based on their potential neuromodulatory activities. Recently, Xia et al. (2021) documented the gut microbiota-mediated protective effect of dietary capsaicin in lipopolysaccharide-induced depression-like behaviors in mice. Dihydrocapsaicin is another capsaicinoid, which has been reported to exert hypothermia-induced neuroprotection in different ischemic stroke animal models (Wu et al., 2017). Similarly, numerous studies have suggested the antidepressant properties of ellagic acid and its derivatives (Mazrooei et al., 2023). Beyond that, all selected compounds followed Lipinski’s rule of five (not more than one violation) and have moderate to high blood-brain barrier permeability (BBB score >1). Therefore, a total of 17 bioactive compounds were selected for subsequent target prediction. Detailed information on the screened compound is listed in Table 2.

**TABLE 2:** Detailed information on the Screened *A. laxiflora* bioactive.

S. No.	PubChem ID	Compound name	OB	DL	BBB	LV
1	5,280,343	Quercetin	0.55	0.52	2.55	0
2	6,475,119	3-Acetylursolic acid	0.85	0.84	1.61	1
3	151,202	3-Acetyloleanolic acid	0.85	0.57	4.61	1
4	91,477	Cholest-4-en-3-one	0.55	0.62	2.79	1
5	2,999,413	Zeranol	0.55	0.5	3.33	0
6	62,453	4-Vinylphenol	0.55	0.29	3.33	0
7	6,452,096	Ethyl iso-allocholate	0.55	0.39	4.08	0
8	1,548,943	Capsaicin	0.55	0.14	2.11	0
9	107,982	Dihydrocapsaicin	0.55	0.1	1.55	0
10	5,319,609	3-O-Methylellagic acid-3′-O-α-rhamnopyranoside	0.55	0.05	2.63	1
11	5,742,590	β-Sitosterol-3-O-β-D-glucopyranoside	0.55	0.5	3.58	1
12	10,140	Glycocholic acid	0.56	0.29	2.73	0
13	538,589	2*H*-Pyran-2-one, tetrahydro-4-hydroxy-6-pentyl	0.55	0.29	4.16	0
14	620,007	4-Fluoro-2-nitroaniline, 5-[4-(pyrrolidin-1-yl)carbonylmethylpiperazin1-yl]	0.55	0.46	4.17	0
15	551,497	D-galactitol, 3,6-anhydro-1,2,4,5-tetra-o-methyl	0.55	0.29	1.08	0
16	253,193	Phaeophorbide A	0.56	0.6	1.93	1
17	14,135,395	Byzantionoside B	0.55	0.6	3.79	0

OB, oral bioavailability; DL, drug-likeness; BBB, blood brain barrier; LV, number of Lipinski’s rule of five violations.

### 3.2 Potential targets of *A. laxiflora* and depression

**FIGURE 3:**
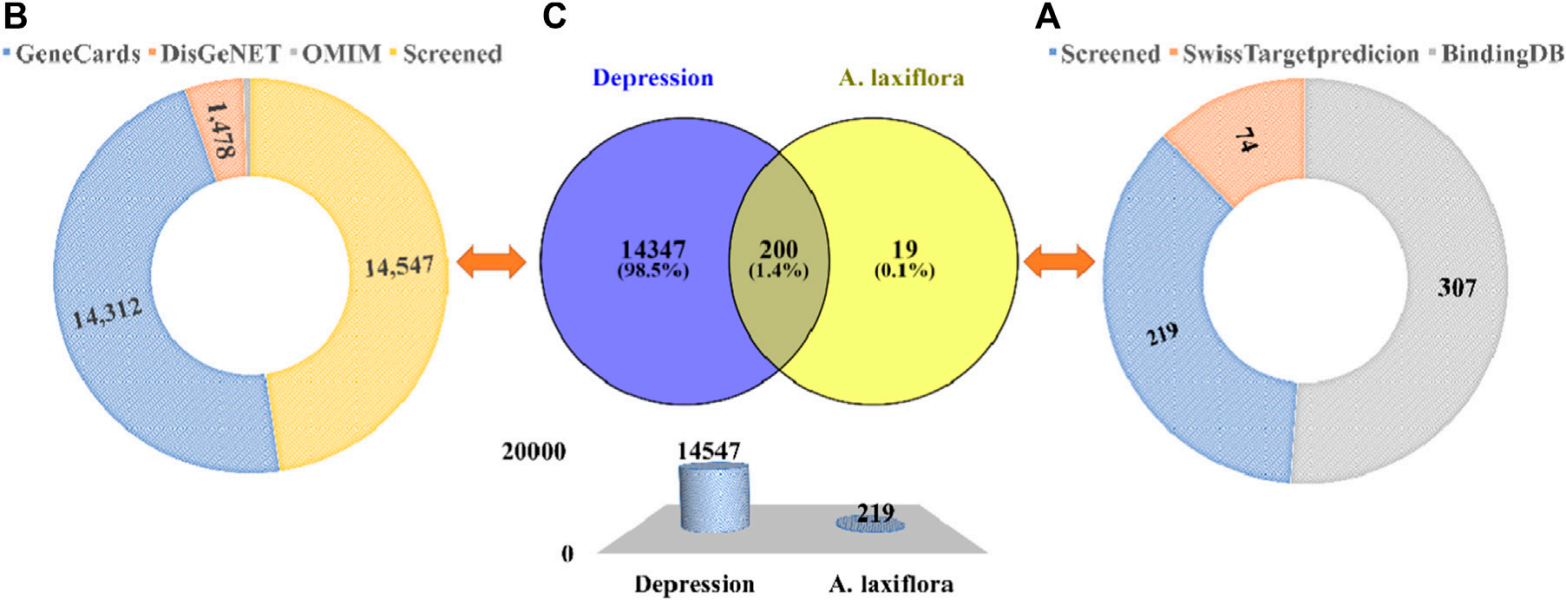
Potential targets of *A. laxiflora* and depression. Sunburst diagram depicting **(A)** Screening of *A. laxiflora* bioactive compounds target and **(B)** Depression related targets from different public databases. **(C)** Venn diagram and bar plot of drug-disease targets.

**FIGURE 4:**
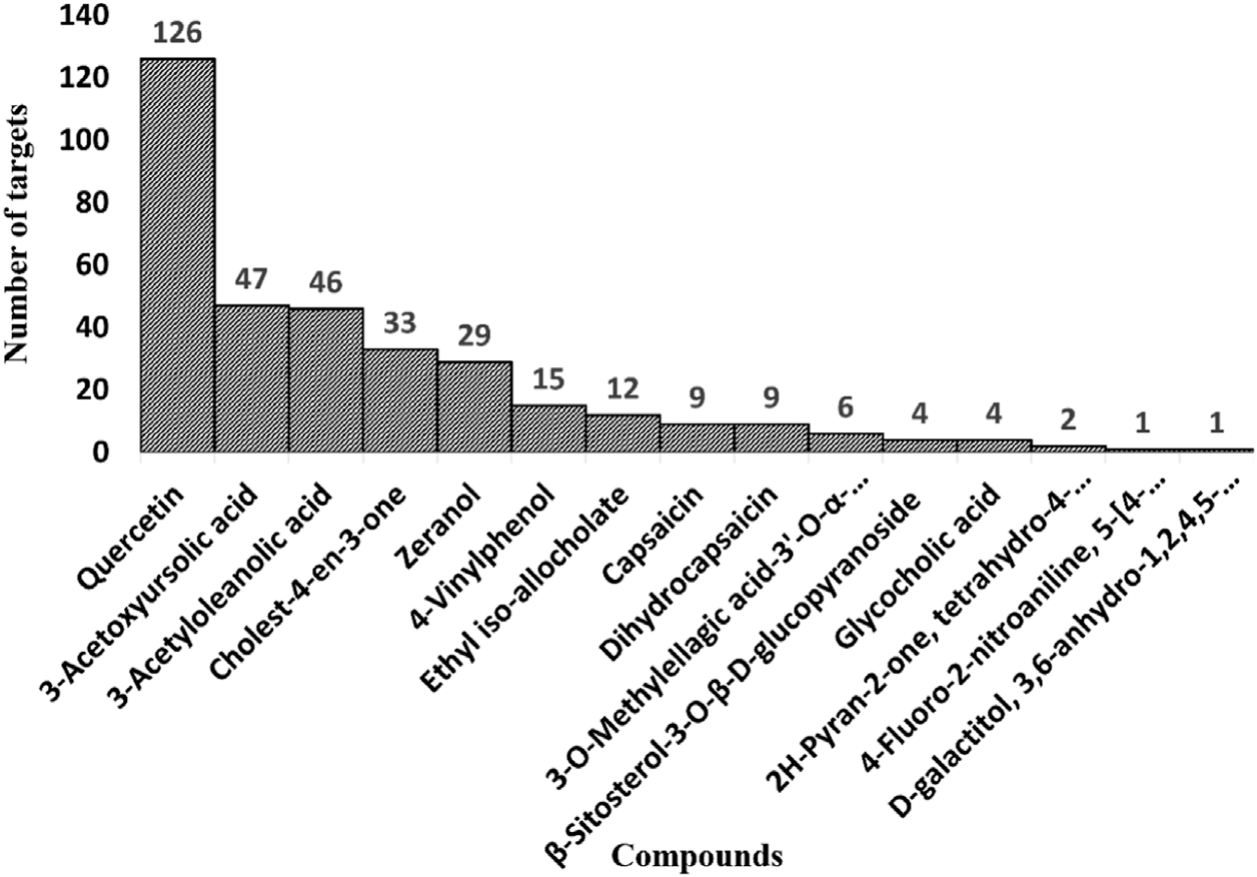
Bar plot of target distribution of various *A. laxiflora* bioactive compounds.

After this, two databases were engaged to collect bioactive compounds-related targets. A total of 77 targets were compiled from the SwissTargetprediction database. Seventy-four remain after deleting the duplicate targets. Among 17 bioactive compounds, only five components yielded predicted targets. A total of 307 targets were predicted by the BindingDB database with a high similarity score of ≥0.70. A total of 189 remain after removing overlapping targets. Among the bioactive compounds, 15 compounds yielded targets, and two predicted none. Both databases’ results were merged, and overlapping targets were removed. Finally, we obtained 219 bioactive targets (Figure 3A). Among the bioactive compounds, quercetin (126), 3-acetylursolic acid (47), 3-acetyloleanolic acid (46), cholest-4-en-3-one (33), and zeranol (29) have the highest number of predicted targets, whereas phaeophorbide A and byzantionoside B have no predicted targets (Figure 4).

Moreover, we searched for depression targets in three public databases: GeneCards, DisGeNET, and OMIM, yielding 14,312, 1,478, and 189 targets, respectively. After removing duplicate values, we obtained 14,547 depression targets (Figure 3B). Both 219 *A. laxiflora* bioactive compounds targets, and 14,547 depression targets were compared by the online Venny2.1 tool to identify intersecting targets as potential targets of *A. laxiflora* for depression via drawing a Venn diagram. Two hundred targets were obtained through the Venn diagram (Figure 3C).

### 3.3 Botanical-BA-TAR network construction

**FIGURE 5:**
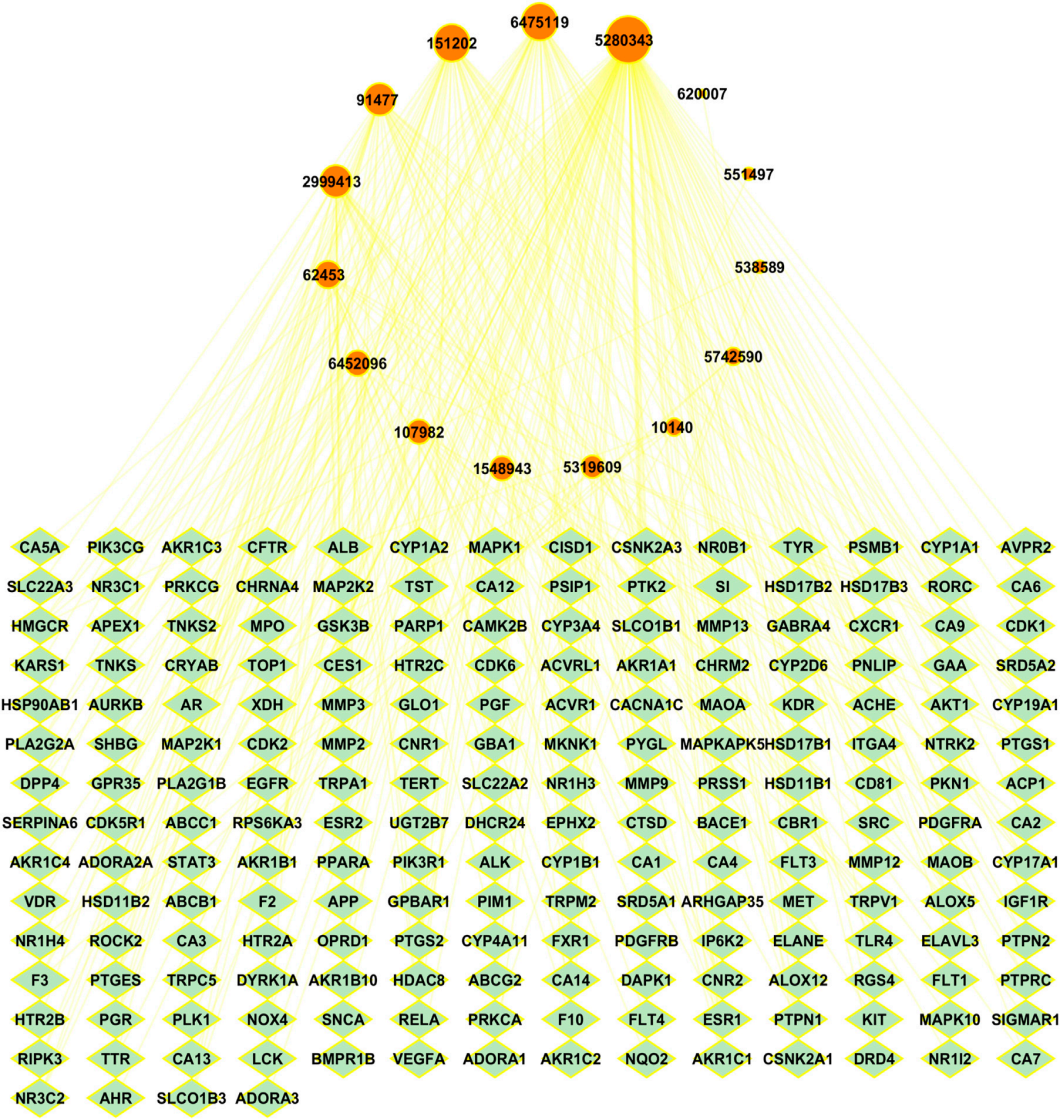
Bioactive-Target network with 216 nodes and 333 edges linking bioactive compounds of *A. laxiflora* with 200 targets of depression. Green colored nodes indicate depression’s potential targets while red colored nodes on top reflects *A. laxiflora’s* 15 bioactives. Larger the node, larger is the degree of bioactive compounds in the network.

To investigate the relationship between *A. laxiflora* bioactive compounds and overlapping targets of depression and *A. laxiflora* bioactive compounds, Cytoscape software was used to build a BA-TAR network. Two hundred potential targets and 15 bioactive compounds of *A. laxiflora* were input into Cytoscape to build a bioactive-target network. The network analyzer was used to identify the nodes and edge count in the network. The constructed network has 216 nodes and 333 edges with an average number of neighbors of 3.08 (Figure 5).

Similarly, the degree of 15 bioactive compounds was also analyzed in the BA-TAR network, indicating multi-component and multi-target characteristics of *A. laxiflora* against depression. As shown in Figure 5, the network of the bioactive compounds was arranged in degree degree-sorted circle layout and size mapped according to the degree analysis. The larger the degree of bioactive compound larger the size of the nodes. The highest degree bearing bioactive compounds of *A. laxiflora* against depression were quercetin, 3-acetylursolic acid, 3-acetyloleanolic acid, cholest-4-en-3-one, and zeranol, which were linked to 116, 43, 42, 34, and 26 genes, respectively (Table 3). It clearly shows the potential of these bioactive compounds in becoming a key component of *A. laxiflora* in treating depressive disorder.

**TABLE 3:** Degree analysis of selected 15 *A. laxiflora* bioactive compounds.

S. No.	Compound	PubChem ID	Class	Degree
1	Quercetin	5,280,343	Flavonoids	116
2	3-Acetylursolic acid	6,475,119	Terpenoids	43
3	3-Acetyloleanolic acid	151,202	Terpenoids	42
4	Cholest-4-en-3-one	91,477	Terpenoids	34
5	Zeranol	2,999,413	Phenolics	26
6	4-Vinylphenol	62,453	Phenolics	16
7	Ethyl iso-allocholate	6,452,096	Terpenoids	12
8	Dihydrocapsaicin	107,982	Alkaloids	10
9	Capsaicin	1,548,943	Alkaloids	10
10	3-O-Methylellagic acid-3′-O-α-rhamnopyranoside	5,319,609	Phenolics	7
11	Glycocholic acid	10,140	Terpenoids	5
12	β-Sitosterol-3-O-β-D-glucopyranoside	5,742,590	Terpenoids	5
13	2H-Pyran-2-one, tetrahydro-4-hydroxy-6-pentyl	538,589	Fatty acids	3
14	D-galactitol, 3,6-anhydro-1,2,4,5-tetra-o-methyl	551,497	Reduced sugar	2
15	4-Fluoro-2-nitroaniline, 5-[4-(pyrrolidin-1-yl)carbonylmethylpiperazin1-yl]	620,007	Alkaloids	2

### 3.4 PPI network construction and identification of depression core targets

**FIGURE 6:**
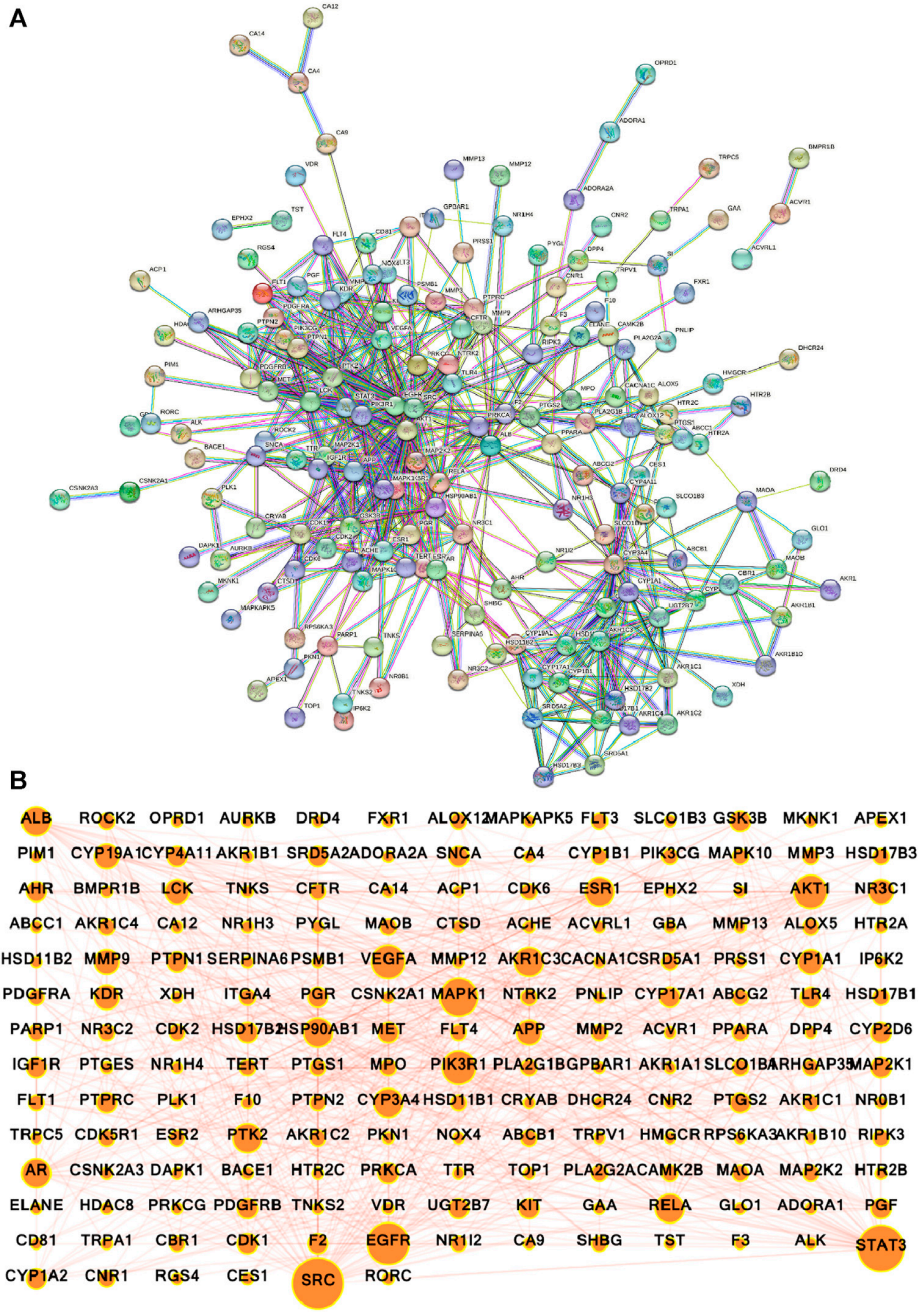
Protein-protein interaction (PPI) network of intersecting targets of *A. laxiflora* and depression. **(A)** STRING database PPI network (confidence score ≥0.70) **(B)** PPI network constructed by Cytoscape v3.9.1. Nodes reflecting targets and edges stand for the interaction between the targets. The larger the nodes, the larger the degree and greater importance in the network.

**FIGURE 7:**
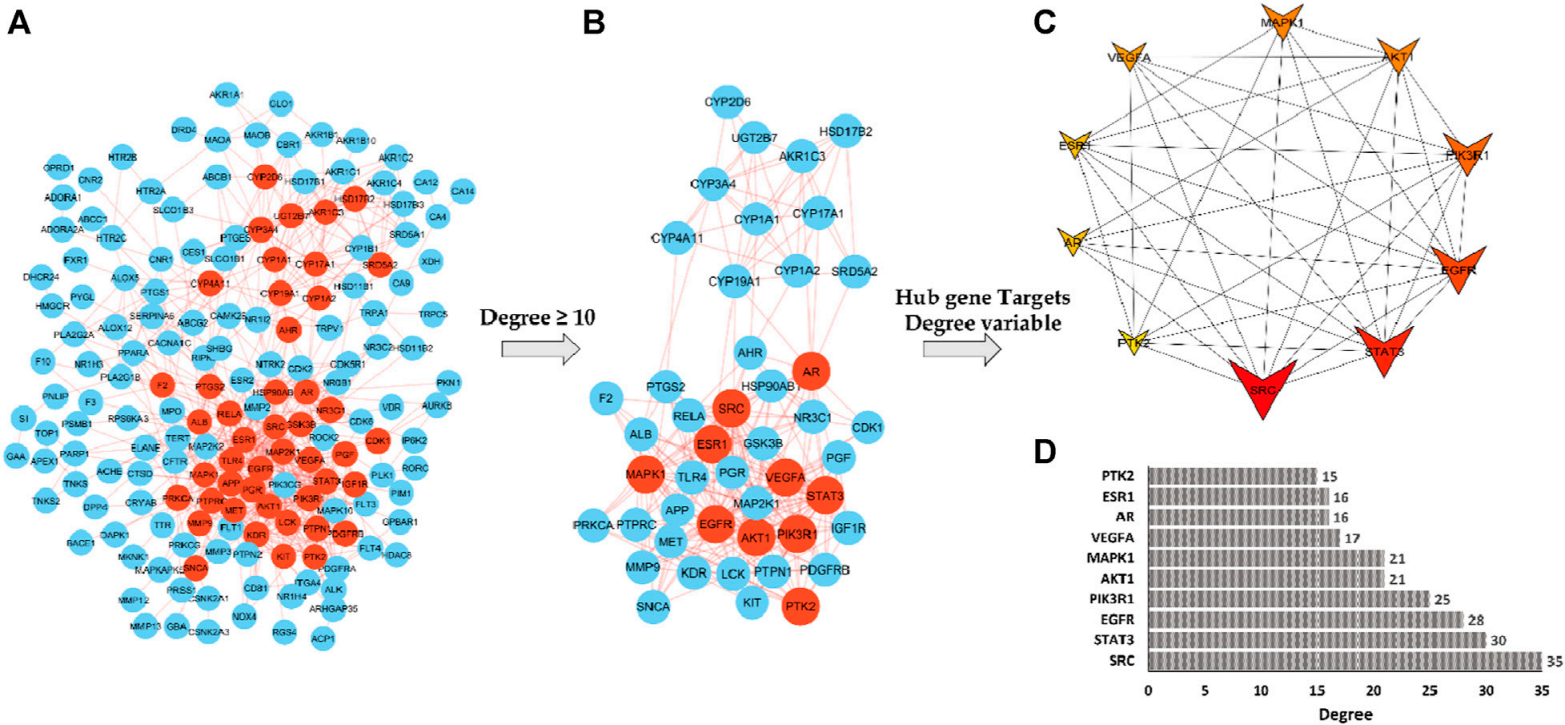
Hub gene target screening process. The red nodes depict the targets that meet the screening criteria **(A,B)**. Top 10 hub genes ranked by degree method **(C)**, Bar chart of degree analysis of top 10 targets **(D)**. Inverted structures depict the top 10 core hub targets. The color and size of the nodes are according to the degree. The larger the size, the larger the degree, and as the degree lowers, the color changes from red to yellow.

The potential gene targets obtained after intersecting bioactive compound targets with the depression gene targets were used to build the PPI network by the STRING database. Figure 6A shows that the network consisted of 200 nodes and 640 edges with an average local clustering coefficient of 0.478 and an average node degree of 6.4. The network results were further imported to Cytoscape for better understanding and visualization of the network. As shown in Figures 6B, 7A, the network visualization demonstrated the presence of 175 nodes and 640 edges with a characteristic path length of 2.611 and an average number of neighbors of 7.314. The node size corresponds to the degree of protein targets in the network. The PPI network was subjected to visual topological analysis using CytoNCA application based on “Degree, Betweenness, and Closeness” centralities. A total of 46 targets were chosen as core targets of depression based on degree ≥10, i.e., two times of median of all nodes (Figure 7B). Among the 46 core targets, SRC (50), STAT3 (41), EGFR (36), MAPK1 (31), PIK3R1 (29), and AKT1 (29) have the greatest degree (Table 4).

**TABLE 4:** Core targets of depression based on degree centrality (DC), betweenness centrality (BC), and closeness centrality (CC).

S. No.	Target	DC	BC	CC	S. No.	Targets	DC	BC	CC
1	SRC	50	5,738.983	0.142041	29	CYP17A1	12	210.5127	0.12766
2	STAT3	41	2,737.239	0.140097	30	PGF	11	17.75029	0.12627
3	EGFR	36	2,739.046	0.140097	31	PRKCA	11	276.6442	0.132018
4	MAPK1	31	2,985.99	0.137767	32	IGF1R	11	80.07095	0.132521
5	PIK3R1	29	622.9562	0.135409	33	SNCA	11	1,121.348	0.130827
6	AKT1	26	1,074.001	0.137767	34	F2	11	1,130.115	0.133641
7	VEGFA	25	1,819.169	0.135093	35	PTPRC	11	425.0942	0.13142
8	ESR1	23	1,430.216	0.136364	36	GSK3B	11	172.4104	0.13024
9	ALB	22	3,340.745	0.1369	37	PGR	10	169.6428	0.133641
10	CYP3A4	22	2,620.855	0.131519	38	CDK1	10	354.2064	0.130729
11	PTK2	21	273.6661	0.13242	39	CYP4A11	10	631.8848	0.127753
12	AR	21	2,463.67	0.13615	40	PTPN1	10	30.16577	0.128603
13	AKR1C3	20	1,317.498	0.128889	41	KIT	10	31.93589	0.129272
14	RELA	20	1,383.181	0.135831	42	HSD17B2	10	48.97945	0.121508
15	HSP90AB1	20	1,284.788	0.13562	43	SRD5A2	10	261.9411	0.124197
16	CYP1A1	17	900.5634	0.128508	44	CYP1A2	10	397.3055	0.125723
17	LCK	16	183.2239	0.132018	45	AHR	10	615.5516	0.132926
18	NR3C1	16	793.8682	0.134156	46	CYP2D6	10	155.5887	0.120666
19	KDR	15	111.483	0.129657					
20	MMP9	15	989.2133	0.133436					
21	CYP19A1	15	1,025.711	0.129368					
22	APP	15	963.9133	0.134156					
23	TLR4	14	548.1858	0.134259					
24	PTGS2	14	1,245.638	0.133333					
25	UGT2B7	14	261.0851	0.122708					
26	PDGFRB	13	21.00354	0.129272					
27	MAP2K1	12	133.7814	0.130142					
28	MET	12	88.36432	0.130533					

The highest degree between the targets suggests the higher interconnectivity and possibility of their involvement in disease development (Alnusaire et al., 2023). Furthermore, the Hub genes were identified using CytoHubba plug-in, with degree technique utilized out of 12 ways of topological analysis. SRC (29), STAT3 (27), EGFR (26), PIK3R1 (21), AKT1 (19), MAPK1(18), AR (16), VEGFA (15), ESR1 (14) and PTK1 (13) were the among the top ten Hub genes with high degree values in the core target sub-network (Figures 7C, D).

### 3.4 GO and KEGG pathway enrichment analysis

**FIGURE 8:**
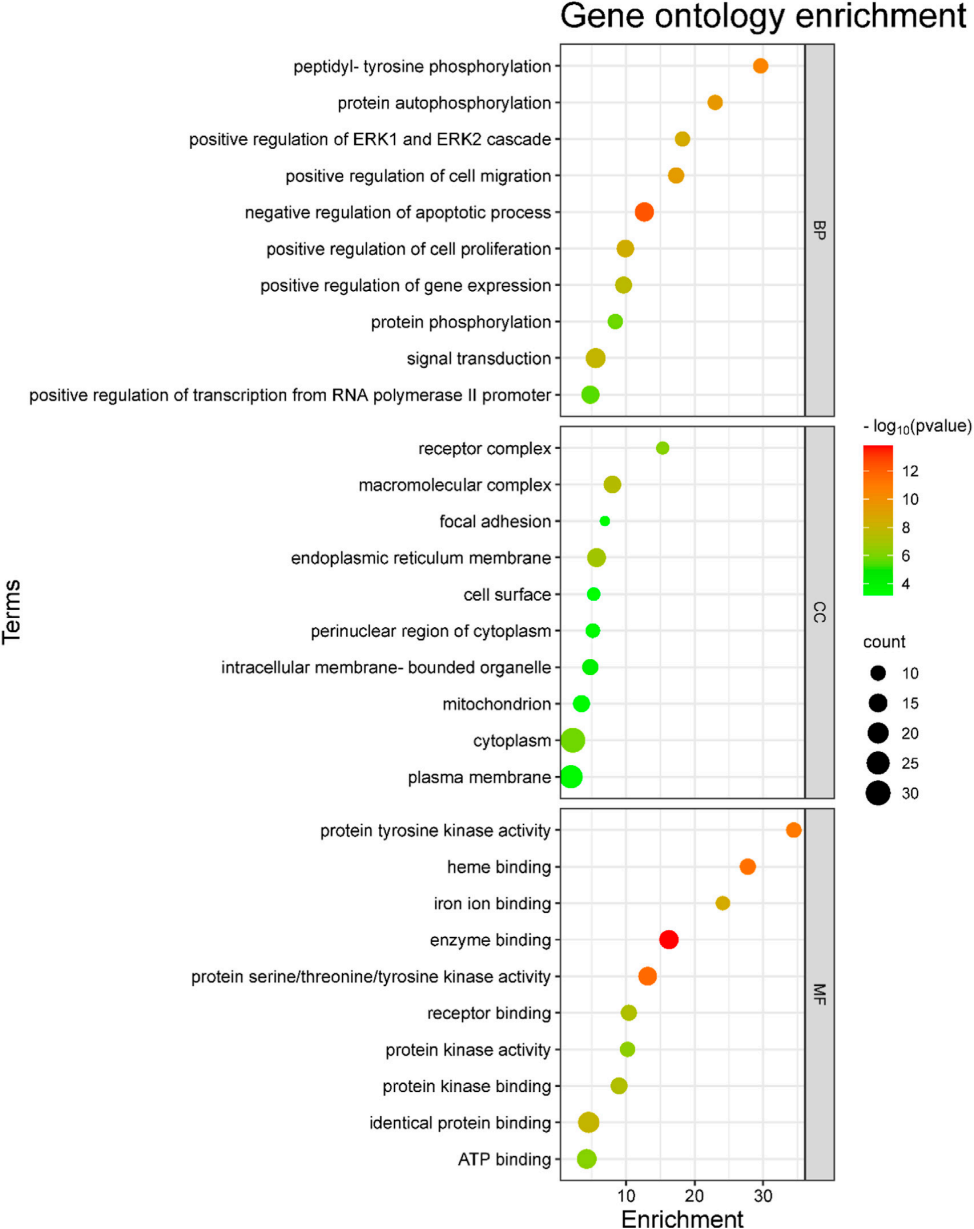
GO analysis of the potential core targets of *A. laxiflora* for depression. The scatter plot of the top 10 biological processes (BP), cellular component (CC), and molecular function analysis of GO enrichment. The enrichment factor and enrichment terms are depicted on the *x*-axis and *y*-axis, respectively.

The 46 antidepressant core targets were subjected to KEGG pathway and GO annotation analysis using the DAVID 2021 functional annotation tool to display the molecular mechanism of *A. laxiflora* in treating depression. From the analyzed targets, significantly enriched (*p* < 0.01) 129 GO terms were obtained, including 82 BP, 14 CC, and 34 MF terms, based on the *p*-value and FDR value. The top ten terms of each BP, CC, and MF were selected to draw the bubble plot, as shown in Figure 8. The five most target-enriched GO BP terms were signal transduction (GO:0007165), negative regulation of apoptotic process (GO:0043066), positive regulation of transcription from RNA polymerase II promoter (GO: 0045944), positive regulation of cell proliferation (GO: 0008284), and positive regulation of gene expression (GO: 0010628). The most affected GO CC entries were cytoplasm (GO: 0005737), plasma membrane (GO: 0005886), endoplasmic reticulum membrane (GO: 0005789), macromolecular complex (GO: 0032991) and mitochondrion (GO: 0005739). The most common GO MF terms were identical protein binding (GO: 0042802), ATP binding (GO: 0005524), enzyme binding (GO: 0019899), protein serine/threonine/tyrosine kinase activity (GO: 0004712), and protein kinase binding (GO: 0019901).

**FIGURE 9:**
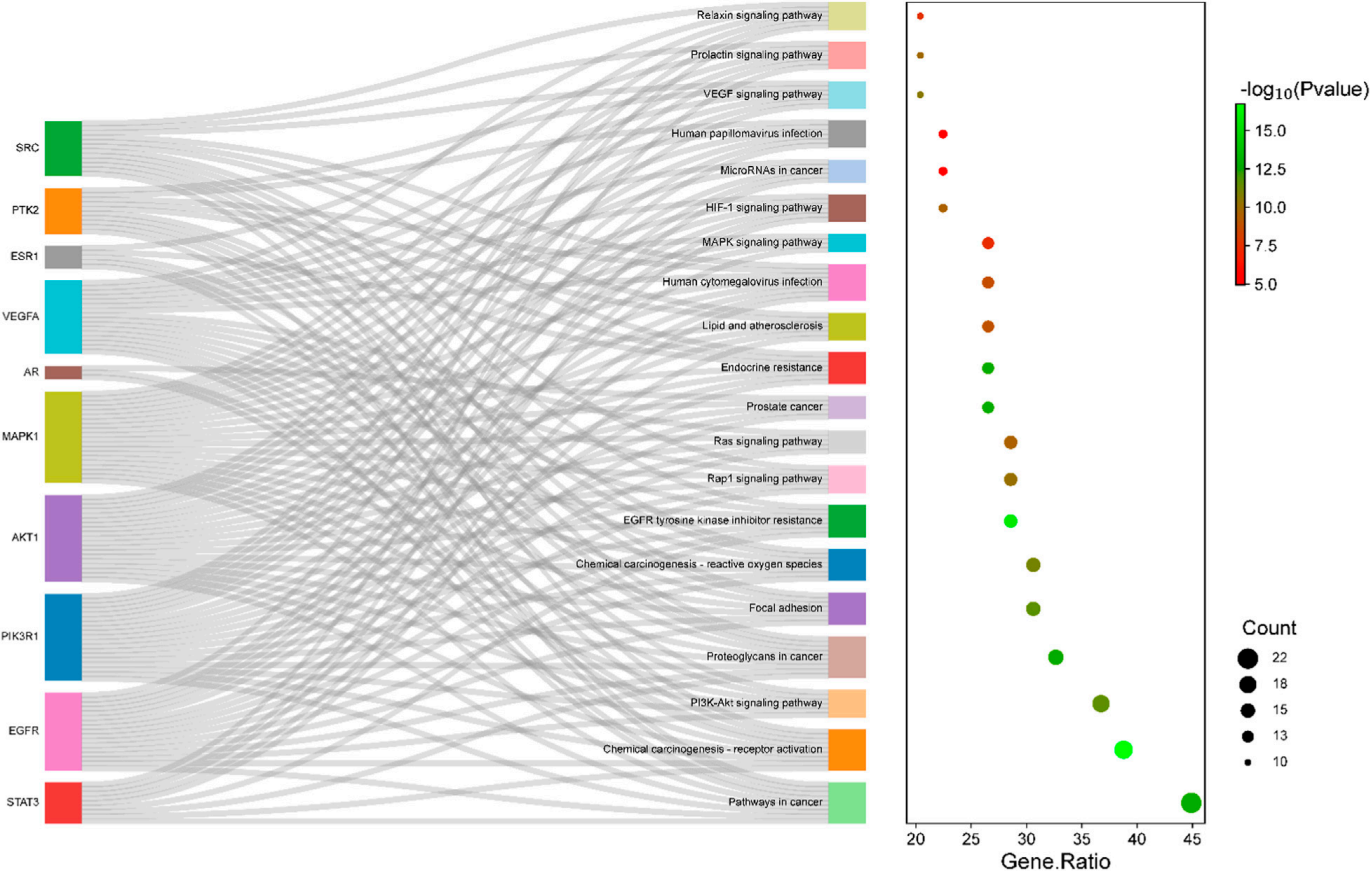
KEGG enrichment pathway. Bubble plot combined with Sankey diagram depicting highly enriched top 20 KEGG pathways and the hub genes within each pathway on the *y*-axis and the gene ratio (*p*-value ≤0.01 and FDR ˂ 0.01) on the *x*-axis.

Furthermore, KEGG enrichment analysis yielded highly enriched 108 pathways. The top 20 significantly enriched (*p* < 0.01) pathways associated with depression were chosen to draw a Sankey diagram with bubble plots (Figure 9). Figure 9 depicts the highly significant enriched top 20 KEGG pathways and the hub genes within each pathway on the *y*-axis and the gene ratio (*p*-value ≤0.01 and FDR ˂ 0.01) on the *x*-axis. Among the top 10 hub gene targets, MAPK1, AKT1, PIK3R1, EGFR, STAT3, and SRC were the most enriched targets in the selected KEGG pathway, suggesting their critical role in the pathogenesis of depression.

**FIGURE 10:**
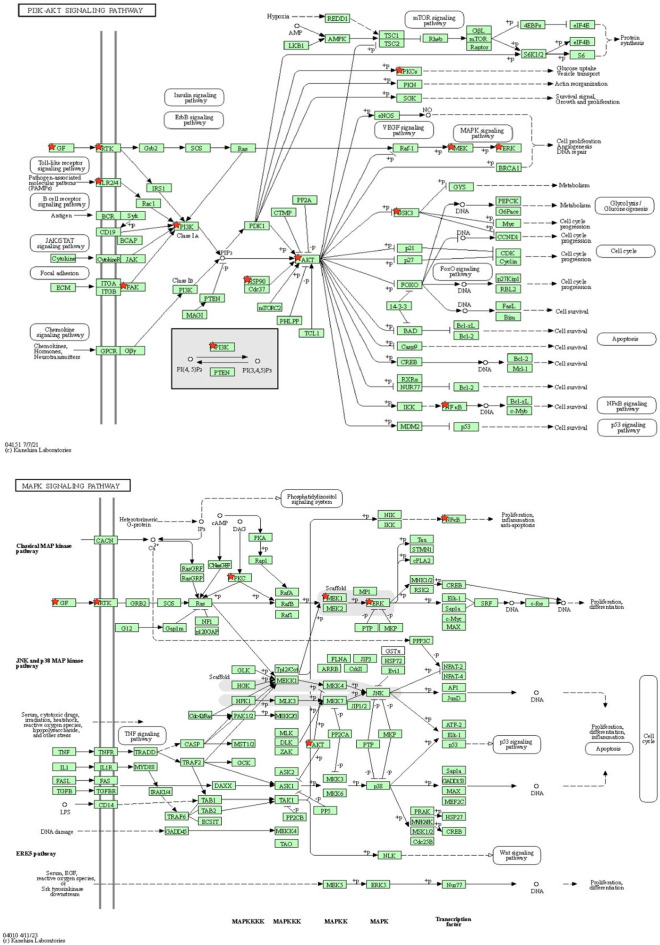
KEGG pathway analysis: **(A)** PI3K-AKT signaling pathway and **(B)** MAPK signaling pathway. The red colored star depicts potential antidepressant targets of *A. laxiflora.*

Among these pathways, the literature review showed a significant association between the PI3K-Akt signaling pathway (hsa04151) and MAPK signaling pathway (hsa04010) in the depression pathophysiology, suggesting targeting of these pathways by *A. laxiflora*’s bioactive compounds as a possible mechanism of action (Su et al., 2017; Li et al., 2023). Notably, most of the PPI network’s hub genes were involved in the PI3K-Akt signaling pathway (hsa04151), highlighting the importance of this pathway for identifying the underlying mechanism of *A. laxiflora* in depression in future exploratory studies. MAPK signaling pathway (hsa04010), Rap1 signaling pathway (hsa04015), Ras signaling pathway (hsa04014), and HIF-1 signaling pathway (hsa04066) were some other prominent enriched pathways that might contribute to the *A. laxiflora* response in depression. Figure 10 demonstrates the PI3K-Akt signaling pathway and MAPK signaling pathways with highlighted potential targets of depression.

### 3.5 BA-TAR-PATH network construction

**FIGURE 11:**
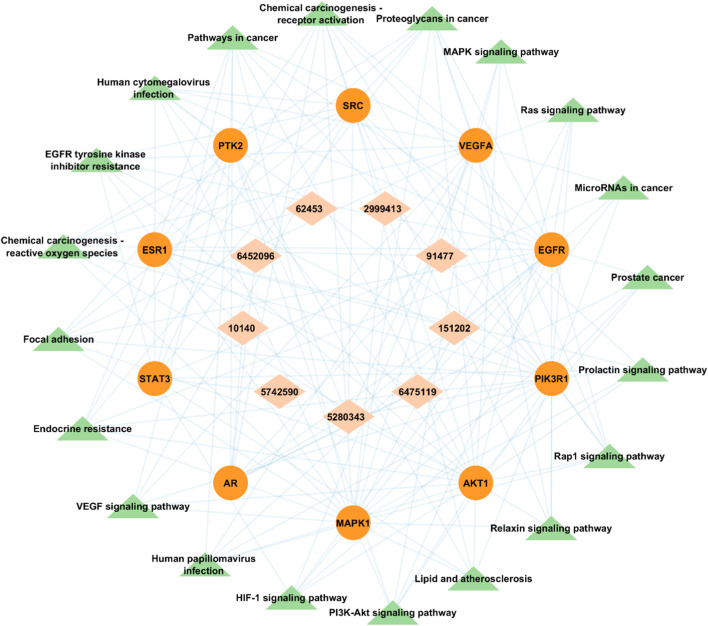
Bioactive- Target-Pathway network in which brick-colored diamonds depict bioactive compounds, red-colored circle nodes represent targets and green-colored triangles represent pathways.

A visual bioactive-target-pathway network was constructed using Cytoscape v3.9.1 to analyze the interaction among bioactive compounds, hub gene targets, and significantly enriched KEGG pathways. The constructed network had 39 nodes (9 bioactive compounds, 10 hub gene targets, and 20 KEGG pathways) and 141 edges (Figure 11). Degree analysis revealed a higher degree of some pathways. Notably, MAPK1, PIK3R1, EGFR, AKT1, and SRC were the essential Hub genes found to be implicated in several pathways, such as PI3K-Akt signaling pathway (hsa04151) and MAPK signaling pathway (hsa04010) among all of the targets studied in the network, which suggests that *A. laxiflora* bioactive compounds may exert antidepressant effect by regulating these targets enriched in reported pathways. Similarly, quercetin, 3-acetylursolic acid, and 3-acetyloleanolic acid had the highest degree in the network, suggesting key ingredients that might play a vital role in the therapeutic effects of *A. laxiflora.*

### 3.6 Molecular docking simulations

By comparing the top ten hub gene targets in the PPI network and the targets involved in the PI3K-Akt signaling pathway (hsa04151) and MAPK signaling pathway (hsa04010), six common proteins (MAPK1, SRC, EGFR, AKT1, STAT3, and PI3KR1) were considered as suitable protein targets for the molecular docking simulation. In general, molecular docking helps to validate the findings from the results of network pharmacology. Hence, molecular docking simulations were carried out for the potential bio-active phytoligands (Quercetin, 3-acetylursolic acid, 3-acetyloleanolic acid, and cholest-4-en-3-one) against each of the identified six protein targets. The binding energy (kcal/mol) of each phytoligand against all the selected targets is presented in Table 5.

**TABLE 5:** The binding energy (kcal/mol) of each phytoligand against all the selected targets.

Phytoconstituent name (PubChem ID)	MAPK1	SRC	EGFR	AKT1	STAT3	PI3KR1
Quercetin (5,280,343)	−8.2	−7.8	−7.8	−9.8	−5.9	−8.3
3-Acetylursolic acid (6,475,119)	−9.2	−9.2	−8.9	−11.5	−6.9	−7.9
3-Acetyloleanolic acid (151,202)	−9.4	−9.1	−8.2	−8.5	−6.8	−8.2
Cholest-4-en-3-one (91,477)	−8.4	−8.1	−8.0	−10.3	−5.8	−8.2

The stability of target-ligand binding depends on the binding energy: the lower the binding energy of the complex, the target-ligand binding interaction will be more stable which is mainly due to the formation of stronger hydrogen bonding interactions between targets and ligands. It is strongly believed that binding energy less than −5 kcal/mol is an indication of better binding affinity between target and ligand, which in turn substantiates better pharmacological activity (Shi et al., 2021). The docking results demonstrated that the binding energies of all the complexes between targets and phytoligands of *A. laxiflora* were determined to be less than −5 kcal/mol.

In particular, phytoligands quercetin and 3-acetylursolic acid had better binding affinities, making more hydrogen bonds to the binding pocket regions of the corresponding target proteins. The quercetin showed better binding energy towards the protein targets AKT1, PI3KR1, and MAPK1 at −9.8 kcal/mol, −8.3 kcal/mol, and −8.2 kcal/mol, respectively. However, quercetin exhibited good equipotent binding affinity against SRC and EGFR at −7.8 kcal/mol, whereas against STAT3, the binding energy is −5.9 kcal/mol indicating appreciable affinity. Among all the phytoligands, the binding energy of 3-acetylursolic acid against the AKT1 target is −11.5 kcal/mol which is the best binding energy that can substantiate and support the better pharmacological profile. Similarly, the same ligand exhibited better equipotent binding affinity against MAPK1, and SRC targets at −9.2 kcal/mol^,^ and with EGFR, it showed a binding energy of −8.9 kcal/mol which is still good. Moreover, it showed appreciable binding affinity against the targets STAT3 and PI3KR1 as evidenced by the binding energy values of −6.9 kcal/mol and −7.9 kcal/mol. The phytoligand 3-acetyloleanolic acid also exhibited better binding affinity towards MAPK1 and SRC with energy of −9.4 kcal/mol and −9.1 kcal/mol, whereas, against the target AKT1, it showed nearly better binding energy of −8.5 kcal/mol. Equipotent binding affinity was also observed for this ligand towards the targets EGFR and PI3KR1 at −8.2 kcal/mol, whereas, with the protein STAT3, the ligand showed appreciable binding energy (−6.8 kcal/mol). The steroid-based phytoligand Cholest-4-en-3-one exhibited the best binding affinity towards AKT1 at an energy of −10.3 kcal/mol, whereas, the same ligand exhibited an almost similar pattern of binding energy between −8.0 kcal/mol to −8.4 kcal/mol with other targets (MAPK1, SRC, EGFR, and PI3KR1). However, the phytoligand Cholest-4-en-3-one showed an appreciable binding with STAT3 protein (−5.8 kcal/mol). To gain a good understanding, the docking simulation results of the key targets and active phytoligands with crucial amino acid residues interacting through intermolecular hydrogen bonding and their bond lengths are collected in Table 6.

**TABLE 6:** Hydrogen bonding interaction between key amino acid residues of targets and active phytoligands with hydrogen bond length in Å.

Target	Quercetin (Å)	3-Acetylursolic acid	3-Acetyloleanolic acid	Cholest-4-en-3-one
MAPK1	Ala-52: 2.8 Å; Gln-105: 2.5 Å; Met-108: 3.1	Ser-153: 2.4 Å	Ser-153: 2.3 Å	—
SRC	Phe-332: 1.9	—	—	—
EGFR	Asp-136: 2.6 Å; Arg-140: 3.2	—	—	—
AKT1	Gln-82: 3.2 Å; Tyr-275: 3.0 Å; Ile-293: 3.1	Tyr-21: 3.1 Å	Tyr-21: 3.3 Å	Arg-276: 3.4 Å
STAT3	Glu-512: 2.0	—	—	—
PI3KR1	Val-812: 2.3 Å and 2.9	—	—	—

Quercetin, a flavonoid group of compounds showed stronger binding against all the studied protein targets, especially against MAPK1, it showed three crucial hydrogen bonding interactions. The carbonyl group and hydroxyl group of benzopyranone moiety of quercetin interacted with the amino acids Met108 and Gln105 through hydrogen bonding at distances of 3.1 Å and 2.5 Å, respectively. The third hydrogen bonding is formed between the hydroxyl group of catechol moiety and Ala52 at 2.8 Å. Further, the quercetin is buried in the binding site amino acid residues such as Leu156, Ser153, Ile31, Asp167, Val39, and Lys54 indicating non-bonding hydrophobic interactions. Against the SRC protein target, the catechol moiety of quercetin exhibited a very strong hydrogen bonding (1.9 Å) with Phe332, whereas, the benzopyranone core moiety of quercetin was surrounded by amino acids such as Gly369, Met366, Val306, Ala318, Glu364, and Phe303. The catechol moiety of quercetin demonstrated two hydrogen bonding interactions with both acidic amino acid Asp136 and basic amino acid Arg140 at distances of 2.5 Å and 3.1 Å, respectively in the target EGFR protein. Hence, the benzopyranone moiety is oriented towards the binding pocket formed by the aliphatic amino acids (Gly18, Leu143, Leu17, Ala42, and Val25). Against the AKT1 protein, the quercetin demonstrated three hydrogen bonding interactions with the amino acids such as Gln82 (3.2 Å), Tyr275 (3.0 Å), and Ile293 (3.1 Å) which indicated a weaker binding affinity for the target-ligand complexes. In the quercetin, the hydroxyl group of catechol moiety has shown a strong hydrogen bonding at 2.0 Å with Glu512 of STAT3 protein. The benzopyranone core moiety of quercetin was surrounded by hydrophilic amino acids such as Gly530, Tyr531, Lys532, and Ile527. Against the PI3KR1 protein, the phytoligand quercetin has shown two significant hydrogen bonding interactions. The hydroxyl and carbonyl oxygen of benzopyranone core of quercetin exhibited hydrogen bonding interactions with the same amino acid Val812 at 2.3 Å and 2.9 Å, respectively.

**FIGURE 12:**
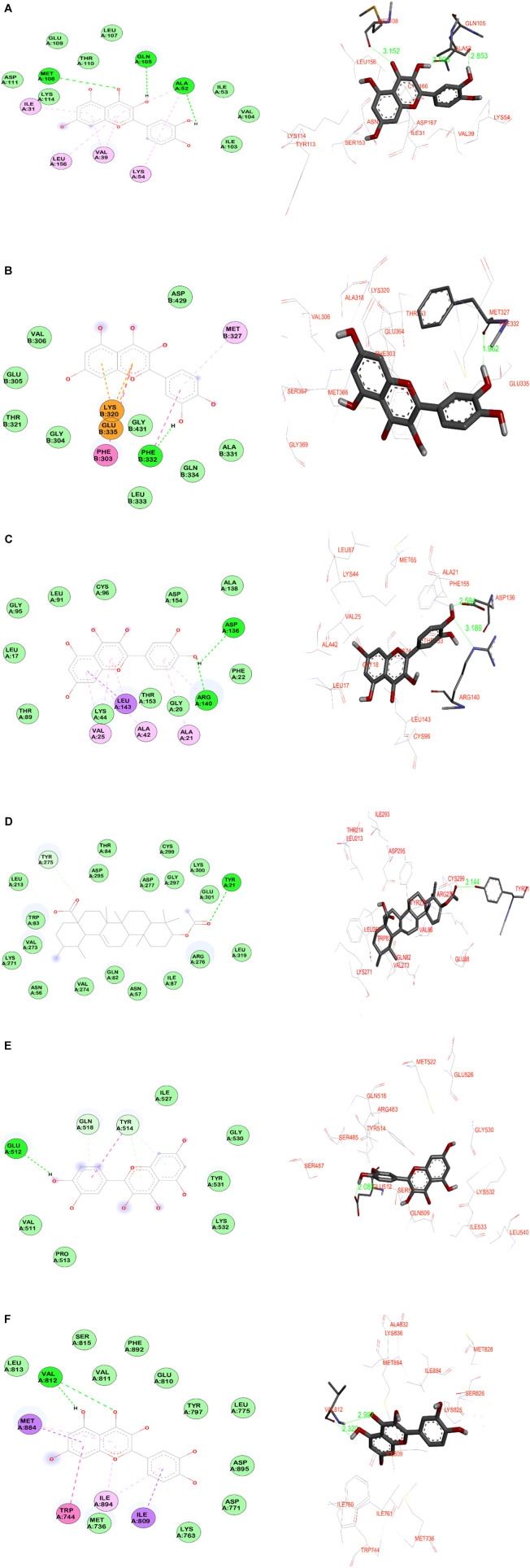
Molecular docking simulation results of the best target-ligand interactions in 2D (left side-wire-frame model of the ligand structure) and 3D (right side-atom colored, thick ball and stick representation) images: **(A)** MAPK1-Quercetin; **(B)** SRC-Quercetin; **(C)** EGFR- Quercetin; **(D)** AKT1-3-acetylursolic acid; **(E)** STAT3- Quercetin; **(F)** PI3KR1-Quercetin. On the 2D figures analysis, green-colored dotted lines indicate hydrogen bonding interactions involving electronegative elements like nitrogen and oxygen atoms; light purple-colored dotted lines indicate π-alkyl interactions; violet-colored dotted lines indicate π-sigma interactions. Light green color amino acids without bonding represent van der Waals interactions, whereas, orange-red color amino acids indicate unfavorable interactions. The light-blue halo surrounding the interacting residues represents the solvent-accessible surface that is proportional to its diameter.

The other potential phytoligand 3-acetylursolic acid interacted well with the protein AKT1. The carbonyl group of the acetyl ester group showed a hydrogen bonding interaction with the hydroxyl group of aromatic amino acid Tyr21 at 3.1 Å. This hydrogen bonding interaction guides the fused pentacyclic structure to orients towards the binding site amino acids such as Glu88, Gln82, Lys271, Leu267, Aspr295, and Arg276. Further, a weak hydrogen bonding is observed between the carbonyl group of carboxylic acid moiety of 3-acetylursolic acid and the hydroxyl group of Tyr275. Against, the MAPK1 protein, the 3-acetylursolic acid has shown one strong hydrogen bond with Ser153 at 2.4 Å 3-acetyloleanolic acid has shown one hydrogen bonding interaction with Ser153 of MAPK1 protein target at 2.3 Å. Against the AKT1 target, this ligand indicated a weak hydrogen bond (3.3 Å) with the aromatic amino acid residue of Tyr21. The ligand cholest-4-en-3-one did not show any significant bonding interactions with almost all the protein targets except the protein AKT1, wherein a weak hydrogen bond (3.4 Å) is observed with the basic amino acid residue Arg276. The data suggested that MAPK1, EGFR, and AKT1 were the major protein targets for achieving the desired pharmacological effect, whereas, quercetin and 3-acetylursolic acid might be predicted as the most active phytoligands of *A. laxiflora* to treat mental depression. The docking simulation resulting in significant molecular interactions of the selected target-ligand complexes is presented in Figure 12.

## 4 Discussion

*A. laxiflora* has been under scrutiny for its impact on neurological disorders such as Parkinson’s disease, Alzheimer’s disease, epilepsy, and anxiety over the last few years (Bum et al., 2009; Nwonu et al., 2018; Ndam Ngoungoure et al., 2019; Ngnameko et al., 2019). In an *in vivo* investigation, *A. laxiflora* leaves aqueous extract at a dose of 120 mg/kg showed a strong protective effect against strychnine-induced seizures. Nwonu et al. (2018) reported significant sedative activity of aqueous and methanolic extract of *A. laxiflora* in mice using a staircase climbing model. Another investigation reported the protective effect of *A. laxiflora* extract against aminochrome-induced toxicity in U373MG and U373MGsiGT6 human astrocytoma cell lines, indicating the potential usefulness of *A. laxiflora* in Parkinson’s disease treatment (Ngoungoure et al., 2019). *A. laxiflora* extracts’ neuro-modulatory potential has been studied using *in vitro* and *in vivo* experimentations. However, information on molecular mechanisms and responsible phytochemicals is still lacking. Depression is another neurological indication against which *A. laxiflora* is under application in folklore medicine (Ngnameko et al., 2019). Network analysis was done to rationalize the antidepressant effect and delineate the molecular mechanism. Herein, we used network pharmacology with molecular docking to elucidate the antidepressant potential of *A. laxiflora* and its mechanism of action. Our study provides a foundation for screening *A. laxiflora* bioactive compounds and suitable target-enriched pathways for future novel therapeutic research against depression.

First, we developed a BA-TAR network of overlapping targets of depression and *A. laxiflora* bioactive compounds to identify the core bioactive compounds having antidepressant potential. Fifteen active components were screened, and the five bioactive compounds identified through the BA-TAR network were quercetin, 3-acetyursolic acid, 3-acetyloleanolic acid, cholest-4-en-3-one, and zeranol, having more targets than rest of the active components. Following the principle of one compound, several targets and several compounds may act on the same target; it is inferred that *A. laxiflora* may act on numerous targets of depression through several compounds.

Multiple studies have suggested the role of quercetin in alleviating anxiety and depression via neuroprotective effects. As a potential antidepressant compound, quercetin is reported to regulate neurotransmitter levels, promote hippocampal neuron regeneration, reduce inflammatory and oxidative stress, and normalize hypothalamus-adrenal axis dysfunction (Chen et al., 2022). Quercetin, the most abundant flavonoid in various fruits and vegetables, exerts good neuroprotective effects due to its ability to cross the blood-brain barrier (Silvestro et al., 2021). Adeoluwa et al. (2023), in a study involving lipopolysaccharide (LPS) induced neuroinflammation in rats, indicated that quercetin could significantly attenuate the expression of inflammasomes, inducible NOS, NF-κB, proinflammatory cytokines, and microglia cells in the hippocampus and prefrontal cortex, thus presenting antidepressant-like property. Similar results were reported by Fang et al. (2019), wherein quercetin could mitigate LPS-induced depression in rats via regulating brain-derived neurotrophic factor (BDNF) related variance expression of Copine 6 and TREM1/2 in the hippocampus and prefrontal cortex. *In vivo*, quercetin demonstrated antioxidant and anti-inflammatory activity in the cerebral cortex and hippocampus by ameliorating the cyclophosphamide-induced oxidative and inflammatory stress via mitigating immunosuppressive indoleamine 2,3-dioxygenase and tryptophane 2,3 dioxygenase activities (Ebokaiwe et al., 2022). Quercetin has also been found to exert antidepressant activity in estrogen receptor-α deficient mice through BDNF-troponin-related kinase B (TrkB)-AKT/ERK1/2 signaling (Wang G. et al., 2021).

3-acetylursolic acid is a natural pentacyclic triterpene ursolic acid derivative in common herbs and medicinal plants reported for various therapeutic effects. Ursolic acid is reported to demonstrate neuroprotection via antioxidant and anti-inflammatory activity in various central nervous system disorders, including anxiety, depression, cognition deficit, brain injury, and cerebral ischemia (Habtemariam, 2019). It has exhibited antidepressant activity in various *in vitro* and *in vivo* studies (González-Cortazar et al., 2013). In an experimental study, ursolic acid prevented chronic unpredictable stress-induced depression-like behavior via modulating Bcl-2/Bax gene expression (Colla et al., 2021). Ursolic acid has various other pharmacological effects, such as anti-cancer, anti-microbial, and anti-diabetic activity. A similar or enhanced therapeutic potential has been reported with other structural ursolic acid derivatives such as amides, ester, and oxadiazole quinolone (Mlala et al., 2019). 3-acetylursolic acid is a 3-acetylated derivative of ursolic acid, which has been reported to demonstrate similar potency and efficacy to ursolic acid when investigated for anti-proliferative and anti-migratory effects on melanoma cells (AlQathama et al., 2020).

Similarly, 3-acetyloleanolic acid is another natural pentacyclic triterpenoid, reported to possess various biological properties such as induction of apoptosis in cancer cell lines, suppressing allergic and atopic contact dermatitis and inflammatory bone loss in mice models (Kim et al., 2016). In an *in vivo* investigation in rats, 3-acetyloleanolic acid was reported to exert a protective effect against hyperlipidemia in non-alcoholic fatty liver disease by activating the AMPK pathway (Ou-Yang et al., 2018). This drug has also been reported to suppress angiogenesis-triggered tumor growth by exhibiting anti-angiogenic and induction of apoptosis in human umbilical vein endothelial cells (Cui et al., 2013). Similar results were obtained in the study reported by (Hwang-Bo et al., 2018), in which 3-acetyloleanolic acid isolated from the seeds of *Vigna sinensis* K. exhibited inhibition of VEGF-A-induced lymph-angiogenesis both *in vitro* and *in vivo* through suppressing VEGF-A-VEGFR-1 and -2 signaling in human lymphatic microvascular endothelial cells (HLMECs) and oral cancer sentinel lymph node animal model (OCSLN). In a recent study, (Hwang-Bo et al., 2020), reported the mechanism of anti-lymph-angiogenic and tumor angiogenesis effect, involving inhibition of activation of downstream signaling factors FAK, AKT, and ERK1/2 associated with an angiopouetin-1-Tie-2 signaling pathway, in both *in vitro* and *in vivo*. Although reported biological activities of 3-acetyloleanolic acid are mostly related to cancer, given its inhibitory effect on AKT and ERK1/2 factors, it can be investigated for antidepressant potential. Our study indicated that this compound might exhibit an antidepressant effect by modulating PI3K-AKT and MAPK signaling pathways.

Additionally, zeranol, an estrogenic lactone derivative, is reported to produce neuroprotective effects in cerebral ischemia-reperfusion rat model mediated by activation of ERK signaling and subsequent inhibition of inflammation and apoptosis (Fleck et al., 2012; Mohamed et al., 2019). Cholest-4-en-3-one is another bioactive highlighted in our study and is a cholesterol and plant sterol metabolite (Nagao et al., 2021). It has been reported to differentiate neural stem cells into dopaminergic neurons via upregulating the TET1 and FoxA2 expression and their binding, indicating potential application as neural stem cell replacement therapy for neurodegenerative diseases (Ye et al., 2020). In a previously reported study, cholest-4-en-3-one induced time-dependent phosphorylation of AKT for inhibiting lung adenocarcinoma metastasis (Ma et al., 2016). Hence, these finding indicates the theoretical basis for these compounds in treating depression alone or synergistic. Our study is probably the first to divulge the potential antidepressant effect of these compounds except quercetin. And they could be the subject of future exploratory studies, resulting in exciting outcomes.

GO enrichment analysis revealed the biological information of antidepressant targets. In the analysis, antidepressant targets of *A. laxiflora* were mainly associated with signal transduction, negative regulation of the apoptotic process, protein serine/threonine/tyrosine kinase activity, and protein kinase binding. Additionally, KEGG analysis disclosed the involvement of targets in multiple depression-related pathways. The putative core targets were significantly enriched in several depression-related pathways, such as the PI3K-Akt signaling pathway, MAPK signaling pathway, Rap1 signaling pathway, Ras signaling pathway, and HIF-1 signaling pathway. The PI3K-AKT and MAPK signaling pathways mainly enriched our core gene targets.

A plethora of evidence suggests that PI3K-AKT signaling is a crucial pathway involved in the pathogenesis of depression (Li et al., 2023). PI3K-Akt signaling regulates various neuronal activities such as synaptic neuroplasticity, cell proliferation, cell migration, and apoptosis (Matsuda et al., 2019). Hence, dysregulation of this signaling is considered to be associated with several mental illnesses, including depression and anxiety. Ye et al. (2019) reported the inhibition of PI3K-Akt signaling as a critical mechanism of action in the neuroprotective effect of sertraline. One of the studies found that catalpol-induced upregulation of the PI3K/Akt/Nrf2/HO-1 signaling pathway may improve hippocampal neuroprotective, neurotrophic, and antioxidant levels in animal models of depression (Wang J. et al., 2021). MAPK signaling is another functionally enriched pathway in the pathobiology of depression. By regulating various downstream mediators such as extracellular signal-regulated kinase (ERK), c-Jun amino-terminal kinase (JNK), and p38 proteins, MAPK signaling may control neuronal cell death and depression-like behavior. Neuroinflammation is one of the aspects of depression mediated by NLRP3 inflammasomes involving the MAPK pathway as one of the downstream signaling in CUMS-induced depression (Su et al., 2017). Moreover, MAPK signaling disruption during depression development is believed to be associated with long-lasting neuroadaptations in the brain, essential for enduring depression and antidepressant efficacy (Wang and Mao, 2019). Polyphenols may ameliorate depression by inhibiting the MAPK signaling pathway-dependent oxidative stress and inflammation in depression (Behl et al., 2022). Recently, Chen et al. (2023) showed the antidepressant potential of saffron essential oil in chronic unpredictable mild stress-induced depression in mice by regulating the MAPK-CREB1-BDNF signaling pathway. In another investigation, paroxetine, in combination with fluorouracil, alleviated depression in colorectal cancer mouse models by inhibiting the IL-22-dependent MAPK signaling pathway (Zhang et al., 2020). In the present investigation, various other pathways were enriched with core targets, such as the HIF-1 signaling pathway, Rap1 signaling pathway, and Ras signaling pathway. Multiple network pharmacology studies have suggested that these pathways have therapeutic efficacy in managing depression. Therefore, the present study indicates that active ingredients of *A. laxiflora* and associated core targets might exert a potent and synergistic effect on depression.

Topological analysis of the bioactive-target-pathway network indicated that MAPK1, PIK3R1, EGFR, AKT1, and SRC were the core targets enriched in crucial signaling pathways associated with treating depression by *A. laxiflora*. Previous studies have suggested downregulation of MAPK1 attenuates depressive-like behaviors and inflammation in CUMS mice (Chang et al., 2021). Moreover, in a recent clinical investigation on 80 patients with major depressive disorder, MAPK1 polymorphism was linked with relapse during antidepressant treatment (Santos et al., 2023). PIK3R1 is an essential member of the PI3K/Akt signaling pathway and is implicated in various cellular events, such as proliferation and apoptosis (Yagci et al., 2019). In a recent investigation, including analysis of microarray profile datasets to recognize CUMS induced differentially expressed genes in pathologically affected brain parts like the anterior cingulate cortex and dentate gyrus, PIK3R1 was one of the critical hub genes identified in the anterior cingulate cortex of the major depressive disorder brain (Wei et al., 2021). Multiple pharmacological network studies have suggested the role of EGFR in major depressive disorder (Zhang T. et al., 2022). EGFR gene upregulation has been linked with multiple cancer types, such as breast cancer, non-small cell lung cancer (NSCLC), and head and neck squamous cell carcinoma (Sekine, 2014).

Furthermore, EGFR amplifications have been linked to various primary tumors of the nervous system, such as glioblastoma and oligodendrogliomas (Alvarez and Bredel, 2013). In a clinical investigation on NSCLC patients to examine the relationship between the severity of the major depressive disorder and EGFR mutation, the results indicated lower depression severity in EGFR-mutated NSCLC patients than with patients harboring wild-EGFR (Jacobs et al., 2017). AKT1 dysregulation is considered an integral component in the pathogenesis of multiple psychiatric disorders (Li et al., 2020). The disease severity, anxiety, and suicidal tendencies in depressive patients were associated with AKT1 polymorphism (Yang et al., 2012). SRC is an important prototype of SRC family kinases (SFKs). It is considered to be involved in multiple cellular processes, including apoptosis, cell proliferation, differentiation, migration, and metabolism (Ortiz et al., 2021; Jain et al., 2023). Previous studies have suggested SRC tyrosine kinase as a potential therapeutic target for various neuroinflammation-related disorders (Yang et al., 2020). Our results indicate the multi-target nature of *A. laxiflora* in treating depression, and these targets might be critical targets for depression.

PI3K-Akt and MAPK signaling pathways were the most prominent pathways highlighted in the present study. Additionally, molecular docking simulation was employed to establish interaction between core targets and high-degree core compounds of *A. laxiflora*. In molecular docking validation, 3-acetylursolic acid and 3-acetyloleanolic acid portrayed excellent binding capacity with all five core targets, such as AKT1, MAPK1, PIK3R1, EGFR, and SRC. The results indicated that 3-acetylursolic acid, 3-acetyloleanolic acid, quercetin, and cholest-4-en-3-one bound stably with the core targets and could be used for treating depression.

## 5 Limitations

The present study is a preliminary attempt to predict the efficacious active compounds, their intended targets, and connected pathways for the treatment of depression, thereby suggesting intriguing theoretical evidence for future experimental and clinical research required for a thorough investigation of *A. laxiflora* potential as an antidepressant medicinal application. Owing to the inherent limitations of network pharmacology, which relies on data mining from different databases. There are possibilities for inconsistencies due to various information resources and experimental results. High throughput techniques like liquid chromatography and mass spectrometry may solve such issues (Batool et al., 2022). The present study also has limitations. The current study is based on traditional claims; no preclinical evidence is available in the public domain. Moreover, this work is carried out *in silico*; no *in vitro* or *in vivo* experiments were conducted. Moreover, only part of the screened bioactive compounds had low to moderate blood-brain barrier permeability. Although based on the results, they exhibit interaction with antidepressant targets as predicted by our network pharmacological investigation, their actual binding sites in the central nervous system are still unclear. Therefore, to validate the findings of our study, detailed preclinical and clinical experiments are required.

## 6 Conclusion

In our study, quercetin, 3-acetylursolic acid, 3-acetyloleanolic acid, cholest-4-en-3-one, and zeranol are the potential active compounds for treating depression according to the analysis done using network pharmacology and docking analysis. *A. laxiflora* treated depression by targeting multiple vital targets such as SRC, STAT3, EGFR, PIK3R1, AKT1, MAPK1, AR, VEGFA, ESR1, and PTK1, and through biological processes such as signaling transduction, negative regulation of the apoptotic process, and positive regulation of transcription from RNA polymerase II promoter. PI3K-AKT signaling and MAPK signaling pathway were the critical signaling pathways utilized by *A. laxiflora* to exert antidepressant action, and MAPK1, PIK3R1, EGFR, AKT1, and SRC were the core targets significantly enriched in these pathways. Molecular docking validation demonstrated that multiple core compounds of *A. laxiflora* could bind stably to the multiple core targets of depression, indicating the suitability of *A. laxiflora* in treating depression. Therefore, the present study provides a novel insight into treating depression in humans using *A. laxiflora*, which exerts multi-component, multi-target, and multi-pathway causal relationships. This underexplored traditional herb may be suitable for in-depth preclinical or clinical investigation.

## Data Availability

The datasets presented in this study can be found in online repositories. The names of the repository/repositories and accession number(s) can be found in the article/supplementary material.

## References

[B1] Abu Nuwar M. Jaradat D. M. M. Chandrasekaran B. Natsheh I. Rasras A. J. Alzubi M. S. H. (2023). Design, synthesis, anticancer screening and molecular modelling studies of novel thiazoles. ChemistrySelect 8, 10.1002/slct.202302319

[B2] Adeoluwa O. A. Olayinka J. N. Adeoluwa G. O. Akinluyi E. T. Adeniyi F. R. Fafure A. (2023). Quercetin abrogates lipopolysaccharide-induced depressive-like symptoms by inhibiting neuroinflammation via microglial NLRP3/NFκB/iNOS signaling pathway. Behav. Brain Res. 450, 114503. 10.1016/j.bbr.2023.114503 37209878

[B3] Alnusaire T. S. Qasim S. Al-Sanea M. M. Hendawy O. Uttra A. M. Ahmed S. R. (2023). Revealing the underlying mechanism of Acacia nilotica against asthma from a systematic perspective: a network pharmacology and molecular docking study. Life 13, 411. 10.3390/life13020411 36836768 PMC9966740

[B4] AlQathama A. Shao L. Bader A. Khondkar P. Gibbons S. M Prieto J. (2020). Differential anti-proliferative and anti-migratory activities of ursolic acid, 3-O-acetylursolic acid and their combination treatments with quercetin on melanoma cells. Biomolecules 10, 894. 10.3390/biom10060894 32545262 PMC7356947

[B5] Alrouji M. Alhumaydhi F. A. Al Abdulmonem W. Sharaf S. E. Shahwan M. Majarisi T. (2023). Identifying β-secretase 1 (BACE1) inhibitors from plant-based compounds: an approach targeting Alzheimer’s therapeutics employing molecular docking and dynamics simulation. Mol. Divers. 25, 10.1007/s11030-023-10726-3 37728805

[B6] Alvarez A. A. Bredel M. (2013). “Chapter 124 - primary tumors of the nervous system,” in, eds. Rimoin D. Pyeritz R., Oxford, UK: Academic Press, .

[B7] Amberger J. S. Bocchini C. A. Schiettecatte F. Scott A. F. Hamosh A. (2015). OMIM.org: online Mendelian Inheritance in Man (OMIM®), an online catalog of human genes and genetic disorders. Nucleic Acids Res. 43, D789–D798. 10.1093/nar/gku1205 25428349 PMC4383985

[B8] Batool S. Javed M. R. Aslam S. Noor F. Javed H. M. F. Seemab R. (2022). Network pharmacology and bioinformatics approach reveals the multi-target pharmacological mechanism of fumaria indica in the treatment of liver cancer. Pharm. (Basel) 15, 654. 10.3390/ph15060654 PMC9229061 35745580

[B9] Behl T. Rana T. Alotaibi G. H. Shamsuzzaman M. Naqvi M. Sehgal A. (2022). Polyphenols inhibiting MAPK signalling pathway mediated oxidative stress and inflammation in depression. Biomed. Pharmacother. 146, 112545. 10.1016/j.biopha.2021.112545 34922112

[B10] Berman H. M. Westbrook J. Feng Z. Gilliland G. Bhat T. N. Weissig H. (2000). The protein data bank. Nucleic Acids Res. 28, 235–242. 10.1093/nar/28.1.235 10592235 PMC102472

[B11] Bum E. N. Taiwe G. S. Nkainsa L. A. Moto F. C. O. Seke Etet P. F. Hiana I. R. (2009). Validation of anticonvulsant and sedative activity of six medicinal plants. Epilepsy Behav. 14, 454–458. 10.1016/j.yebeh.2008.12.022 19162225

[B12] Chandran U. Patwardhan B. (2017). Network ethnopharmacological evaluation of the immunomodulatory activity of Withania somnifera. J. Ethnopharmacol. 197, 250–256. 10.1016/j.jep.2016.07.080 27487266

[B13] Chang J. Zhang Y. Shen N. Zhou J. Zhang H. (2021). MiR-129-5p prevents depressive-like behaviors by targeting MAPK1 to suppress inflammation. Exp. Brain Res. 239, 3359–3370. 10.1007/s00221-021-06203-8 34482419

[B14] Chen S. Tang Y. Gao Y. Nie K. Wang H. Su H. (2022). Antidepressant potential of quercetin and its glycoside derivatives: a comprehensive review and update. Front. Pharmacol. 13. Available at:. 10.3389/fphar.2022.865376 PMC9024056 35462940

[B15] Chen Z. Gu J. Lin S. Xu Z. Xu H. Zhao J. (2023). Saffron essential oil ameliorates CUMS-induced depression-like behavior in mice via the MAPK-CREB1-BDNF signaling pathway. J. Ethnopharmacol. 300, 115719. 10.1016/j.jep.2022.115719 36126781

[B16] Cheng T. Pan Y. Hao M. Wang Y. Bryant S. H. (2014). PubChem applications in drug discovery: a bibliometric analysis. Drug Discov. Today 19, 1751–1756. 10.1016/j.drudis.2014.08.008 25168772 PMC4252728

[B17] Colla A. R. S. Pazini F. L. Lieberknecht V. Camargo A. Rodrigues A. L. S. (2021). Ursolic acid abrogates depressive-like behavior and hippocampal pro-apoptotic imbalance induced by chronic unpredictable stress. Metab. Brain Dis. 36, 437–446. 10.1007/s11011-020-00658-4 33394285

[B18] Cui E.-J. Hwang-Bo J. Park J.-H. Baek N.-I. Kim J. Hong S. G. (2013). 3-O-Acetyloleanolic acid exhibits anti-angiogenic effects and induces apoptosis in human umbilical vein endothelial cells. Biotechnol. Lett. 35, 1807–1815. 10.1007/s10529-013-1266-7 23801119

[B19] Cui Y. Wang H. Wang D. Mi J. Chen G. Li F. (2021). Network pharmacology analysis on the mechanism of Huangqi Sijunzi decoction in treating cancer-related fatigue. J. Healthc. Eng. 2021, 9780677. 10.1155/2021/9780677 35154614 PMC8837426

[B20] Daina A. Michielin O. Zoete V. (2017). SwissADME: a free web tool to evaluate pharmacokinetics, drug-likeness and medicinal chemistry friendliness of small molecules. Sci. Rep. 7, 42717. 10.1038/srep42717 28256516 PMC5335600

[B21] Ebokaiwe A. P. Ushang O. R. Ogunwa T. H. Kikiowo B. Olusanya O. (2022). Quercetin attenuates cyclophosphamide induced-immunosuppressive indoleamine 2,3-dioxygenase in the hippocampus and cerebral cortex of male Wister rats. J. Biochem. Mol. Toxicol. 36, e23179. 10.1002/jbt.23179 35906875

[B22] Esan O. Esan A. (2016). Epidemiology and burden of bipolar disorder in Africa: a systematic review of data from Africa. Soc. Psychiatry Psychiatr. Epidemiol. 51, 93–100. 10.1007/s00127-015-1091-5 26155900

[B23] Fang K. Li H.-R. Chen X.-X. Gao X.-R. Huang L.-L. Du A.-Q. (2019). Quercetin alleviates LPS-induced depression-like behavior in rats via regulating BDNF-related imbalance of copine 6 and TREM1/2 in the Hippocampus and PFC. Front. Pharmacol. 10, 1544. 10.3389/fphar.2019.01544 32009956 PMC6978986

[B24] Fleck S. C. Hildebrand A. A. Pfeiffer E. Metzler M. (2012). Catechol metabolites of zeranol and 17β-estradiol: a comparative *in vitro* study on the induction of oxidative DNA damage and methylation by catechol-O-methyltransferase. Toxicol. Lett. 210, 9–14. 10.1016/j.toxlet.2012.01.010 22285433

[B25] Gao Y. Gao L. Tian J.-S. Qin X.-M. Zhang X. (2018). A network pharmacology approach to decipher the mechanisms of anti-depression of Xiaoyaosan formula. World J. Tradit. Chin. Med. 4, 147–162. 10.4103/wjtcm.wjtcm_20_18

[B26] Gbadamosi I. T. Henneh I. T. Aluko O. M. Yawson E. O. Fokoua A. R. Koomson A. (2022). Depression in sub-saharan Africa. IBRO Neurosci. Rep. 12, 309–322. 10.1016/j.ibneur.2022.03.005 35746974 PMC9210463

[B27] Gfeller D. Grosdidier A. Wirth M. Daina A. Michielin O. Zoete V. (2014). SwissTargetPrediction: a web server for target prediction of bioactive small molecules. Nucleic Acids Res. 42, W32–W38. 10.1093/nar/gku293 24792161 PMC4086140

[B28] Gilson M. K. Liu T. Baitaluk M. Nicola G. Hwang L. Chong J. (2016). BindingDB in 2015: a public database for medicinal chemistry, computational chemistry, and systems pharmacology. Nucleic Acids Res. 44, D1045–D1053. 10.1093/nar/gkv1072 26481362 PMC4702793

[B29] González-Cortazar M. Maldonado-Abarca A. M. Jiménez-Ferrer E. Marquina S. Ventura-Zapata E. Zamilpa A. (2013). Isosakuranetin-5-O-rutinoside: a new flavanone with antidepressant activity isolated from Salvia elegans Vahl. Molecules 18, 13260–13270. 10.3390/molecules181113260 24165584 PMC6270368

[B30] Guo W. Huang J. Wang N. Tan H.-Y. Cheung F. Chen F. (2019). Integrating network pharmacology and pharmacological evaluation for deciphering the action mechanism of herbal formula Zuojin pill in suppressing hepatocellular carcinoma. Front. Pharmacol. 10, 1185. Available at:. 10.3389/fphar.2019.01185 31649545 PMC6795061

[B31] Gupta M. Lee H. J. Barden C. J. Weaver D. F. (2019). The blood-brain barrier (BBB) score. J. Med. Chem. 62, 9824–9836. 10.1021/acs.jmedchem.9b01220 31603678

[B32] Habtemariam S. (2019). Antioxidant and anti-inflammatory mechanisms of neuroprotection by ursolic acid: addressing brain injury, cerebral ischemia, cognition deficit, anxiety, and depression. Oxid. Med. Cell. Longev. 2019, 8512048. 10.1155/2019/8512048 31223427 PMC6541953

[B33] He J. Han D. Jia C. Xie J. Zhu F. Wei J. (2023). Integrating network pharmacology, molecular docking and pharmacological evaluation for exploring the polyrhachis vicina rogers in ameliorating depression. Drug Des. devel. Ther. 17, 717–735. 10.2147/DDDT.S399183 PMC10010188 36923105

[B34] He Q. Chen X. Liu J. Li C. Xing H. Shi Y. (2021). Combining network pharmacology with molecular docking for mechanistic research on thyroid dysfunction caused by polybrominated diphenyl ethers and their metabolites. Biomed. Res. Int. 2021, 2961747. 10.1155/2021/2961747 34840968 PMC8613503

[B35] Hwang-Bo J. Bae M. G. Park J. H. Chung I. S. (2018). 3-O-Acetyloleanolic acid inhibits VEGF-A-induced lymphangiogenesis and lymph node metastasis in an oral cancer sentinel lymph node animal model. BMC Cancer 18, 714. 10.1186/s12885-018-4630-0 29976150 PMC6034267

[B36] Hwang-Bo J. Park J. H. Chung I. S. (2020). 3-O-Acetyloleanolic acid inhibits angiopoietin-1-induced angiogenesis and lymphangiogenesis via suppression of angiopoietin-1/Tie-2 signaling. Phytother. Res. 34, 359–367. 10.1002/ptr.6526 31680342

[B37] Ibrahim R. S. El-Banna A. A. (2021). Network pharmacology-based analysis for unraveling potential cancer-related molecular targets of Egyptian propolis phytoconstituents accompanied with molecular docking and *in vitro* studies. RSC Adv. 11, 11610–11626. 10.1039/d1ra01390d 35423607 PMC8695995

[B38] Jacobs J. M. Traeger L. Eusebio J. Simon N. M. Sequist L. V. Greer J. A. (2017). Depression, inflammation, and epidermal growth factor receptor (EGFR) status in metastatic non-small cell lung cancer: a pilot study. J. Psychosom. Res. 99, 28–33. 10.1016/j.jpsychores.2017.05.009 28712427

[B39] Jain N. K. Agrawal A. Kulkarni G. T. Tailang M. (2022a). Molecular docking study on phytoconstituents of traditional ayurvedic drug tulsi (Ocimum sanctum linn.) against COVID-19 Mpro enzyme: an *in silico* study. Int. J. Pharm. Pharm. Sci. 14, 44–50. 10.22159/ijpps.2022v14i4.43181

[B41] Jain N. K. Tailang M. Jain H. K. Chandrasekaran B. Sahoo B. M. Subramanian A. (2023). Therapeutic implications of current Janus kinase inhibitors as anti-COVID agents: a review. Front. Pharmacol. 14, 1135145. Available at:. 10.3389/fphar.2023.1135145 37021053 PMC10067607

[B42] Jain N. K. Tailang M. Kumar S. Chandrasekaran B. Alghazwani Y. Chandramoorthy H. C. (2022b). Appraising the therapeutical potentials of *Alchornea laxiflora* (Benth) Pax & K. Hoffm, an underexplored medicinal herb: a systematic review. Front. Pharmacol. 13, 958453. 10.3389/fphar.2022.958453 36545314 PMC9761395

[B43] James T. Hsieh M. L. Knipling L. Hinton D. (2015). Determining the architecture of a protein-DNA complex by combining FeBABE cleavage analyses, 3-D printed structures, and the ICM Molsoft program. Methods Mol. Biol. 1334, 29–40. 10.1007/978-1-4939-2877-4_3 26404142

[B44] Jiao X. Sherman B. T. Huang D. W. Stephens R. Baseler M. W. Lane H. C. (2012). DAVID-WS: a stateful web service to facilitate gene/protein list analysis. Bioinformatics 28, 1805–1806. 10.1093/bioinformatics/bts251 22543366 PMC3381967

[B45] Jiashuo W. U. Fangqing Z. Zhuangzhuang L. I. Weiyi J. Yue S. (2022). Integration strategy of network pharmacology in Traditional Chinese Medicine: a narrative review. J. Tradit. Chin. Med. = Chung i tsa chih ying wen pan 42, 479–486. 10.19852/j.cnki.jtcm.20220408.003 35610020 PMC9924699

[B46] Kanehisa M. Goto S. (2000). KEGG: kyoto encyclopedia of genes and genomes. Nucleic Acids Res. 28, 27–30. 10.1093/nar/28.1.27 10592173 PMC102409

[B47] Kim E. Noh K. Lee S. J. Shin B. Hwang J. T. Lee S. W. (2016). Simultaneous determination of 3-O-acetyloleanolic acid and oleanolic acid in rat plasma using liquid chromatography coupled to tandem mass spectrometry. J. Pharm. Biomed. Anal. 118, 96–100. 10.1016/j.jpba.2015.10.030 26520257

[B48] Li D. Liu L. Yang S. Xing Y. Pan L. Zhao R. (2021). Exploring the therapeutic mechanisms of huzhang–shanzha herb pair against coronary heart disease by network pharmacology and molecular docking. Evidence-Based Complement. Altern. Med. 2021, 5569666. 10.1155/2021/5569666 PMC8651359 34887932

[B49] Li W. Liu X. Qiao H. (2020). Downregulation of hippocampal SIRT6 activates AKT/CRMP2 signaling and ameliorates chronic stress-induced depression-like behavior in mice. Acta Pharmacol. Sin. 41, 1557–1567. 10.1038/s41401-020-0387-5 32265492 PMC7921578

[B50] Li X. Teng T. Yan W. Fan L. Liu X. Clarke G. (2023). AKT and MAPK signaling pathways in hippocampus reveals the pathogenesis of depression in four stress-induced models. Transl. Psychiatry 13, 200. 10.1038/s41398-023-02486-3 37308476 PMC10261007

[B51] Li X. Wei S. Niu S. Ma X. Li H. Jing M. (2022). Network pharmacology prediction and molecular docking-based strategy to explore the potential mechanism of Huanglian Jiedu Decoction against sepsis. Comput. Biol. Med. 144, 105389. 10.1016/j.compbiomed.2022.105389 35303581

[B52] Lin H. Y. Tsai J. C. Wu L. Y. Peng W. H. (2020). Reveals of new candidate active components in hemerocallis radix and its anti-depression action of mechanism based on network pharmacology approach. Int. J. Mol. Sci. 21, 1868. 10.3390/ijms21051868 32182911 PMC7084327

[B53] Ma J. Fu G. Wu J. Han S. Zhang L. Yang M. (2016). 4-cholesten-3-one suppresses lung adenocarcinoma metastasis by regulating translocation of HMGB1, HIF1α and Caveolin-1. Cell Death Dis. 7, e2372. 10.1038/cddis.2016.281 27899819 PMC5059879

[B54] Maina M. B. Ahmad U. Ibrahim H. A. Hamidu S. K. Nasr F. E. Salihu A. T. (2020). 20 years of african neuroscience: waking a sleeping giant. Cold Spring Harbor Laboratory, Cold Spring Harbor, NY, USA, 10.1101/2020.06.03.131391

[B55] Matsuda S. Ikeda Y. Murakami M. Nakagawa Y. Tsuji A. Kitagishi Y. (2019). Roles of PI3K/AKT/GSK3 pathway involved in psychiatric illnesses. Dis. Basel, Switz. 7, 22. 10.3390/diseases7010022 PMC6473240 30781836

[B56] Mazrooei Z. Dehkordi H. T. Shahraki M. H. Lorigooini Z. Zarean E. Amini-Khoei H. (2023). Ellagic acid through attenuation of neuro-inflammatory response exerted antidepressant-like effects in socially isolated mice. Heliyon 9, e15550. 10.1016/j.heliyon.2023.e15550 37151621 PMC10161705

[B57] Mlala S. Oyedeji A. O. Gondwe M. Oyedeji O. O. (2019). Ursolic acid and its derivatives as bioactive agents. Molecules 24, 2751. 10.3390/molecules24152751 31362424 PMC6695944

[B58] Mohamed S. K. Ahmed A. A. E. Elmorsy E. M. Nofal S. (2019). ERK activation by zeranol has neuroprotective effect in cerebral ischemia reperfusion. Life Sci. 227, 137–144. 10.1016/j.lfs.2019.04.035 31005550

[B59] Mooers B. H. M. (2020). Shortcuts for faster image creation in PyMOL. Protein Sci. 29, 268–276. 10.1002/pro.3781 31710740 PMC6933860

[B60] Morris G. M. Huey R. Lindstrom W. Sanner M. F. Belew R. K. Goodsell D. S. (2009). AutoDock4 and AutoDockTools4: automated docking with selective receptor flexibility. J. Comput. Chem. 30, 2785–2791. 10.1002/jcc.21256 19399780 PMC2760638

[B61] Nagao K. Inoue N. Suzuki K. Shimizu T. Yanagita T. (2021). The cholesterol metabolite cholest-5-en-3-one alleviates hyperglycemia and hyperinsulinemia in obese (db/db) mice. Metabolites 12, 26. 10.3390/metabo12010026 35050148 PMC8779233

[B62] Najmi A. Alam M. S. Thangavel N. Taha M. M. E. Meraya A. M. Albratty M. (2023). Synthesis, molecular docking, and *in vivo* antidiabetic evaluation of new benzylidene-2,4-thiazolidinediones as partial PPAR-γ agonists. Sci. Rep. 13, 19869. 10.1038/s41598-023-47157-x 37963936 PMC10645977

[B63] Ndam Ngoungoure V. L. Mfotie Njoya E. Ngamli Fewou S. Fils Ella A. McGaw L. J. Moundipa P. F. (2019). Acetylcholinesterase inhibitory, anti-inflammatory and antioxidant properties of some Cameroonian medicinal plants used to treat some neurological disorders. Investig. Med. Chem. Pharmacol. 2, 1–13. 10.31183/imcp.2019.00033

[B64] Nelson V. K. Nuli M. V. Mastanaiah J. Saleem T. S. M. Birudala G. Jamous Y. F. (2023). Reactive oxygen species mediated apoptotic death of colon cancer cells: therapeutic potential of plant derived alkaloids. Front. Endocrinol. (Lausanne). 14, 1201198. Available at:. 10.3389/fendo.2023.1201198 37560308 PMC10408138

[B65] Ngnameko C. R. Njayou F. N. Fowora M. Nemg F. B. S. Moundipa Fewou P. Smith S. I. (2019). Inhibitory effect of medicinal plants from Cameroon on the growth and adhesion of *Helicobacter pylori* . Eur. J. Integr. Med. 30, 100957. 10.1016/j.eujim.2019.100957

[B66] Ngoungoure V. L. N. Muñoz P. Tizabi Y. Valdes R. Moundipa P. F. Segura-Aguilar J. (2019). Protective effects of crude plant extracts against aminochrome-induced toxicity in human astrocytoma cells: implications for Parkinson’s disease. Clin. Pharmacol. Transl. Med. 3, 125–133. Available at: http://www.ncbi.nlm.nih.gov/pubmed/31321384%0A. 31321384 PMC6639011

[B67] Nwonu C. Ilesanmi O. Agbedahunsi J. Nwonu P. (2018). Anti-anxiety activities of the aqueous and methanol extracts of Alchornea laxiflora in albino mice. Sci. Res. J. 6, 69–77. 10.29322/ijsrp.8.5.2018.p7781

[B68] Oladunmoye M. K. Kehinde F. Y., and (2011). Ethnobotanical survey of medicinal plants used in treating viral infections among Yoruba tribe of South Western Nigeria. Afr. J. Microbiol. Res. 5, 2991–3004. 10.5897/AJMR10.004

[B69] Ortiz M. A. Mikhailova T. Li X. Porter B. A. Bah A. Kotula L. (2021). Src family kinases, adaptor proteins and the actin cytoskeleton in epithelial-to-mesenchymal transition. Cell Commun. Signal. 19, 67. 10.1186/s12964-021-00750-x 34193161 PMC8247114

[B70] Ou-Yang Q. Xuan C.-X. Wang X. Luo H.-Q. Liu J.-E. Wang L.-L. (2018). 3-Acetyl-oleanolic acid ameliorates non-alcoholic fatty liver disease in high fat diet-treated rats by activating AMPK-related pathways. Acta Pharmacol. Sin. 39, 1284–1293. 10.1038/aps.2017.142 29345253 PMC6289400

[B71] Piñero J. Bravo À. Queralt-Rosinach N. Gutiérrez-Sacristán A. Deu-Pons J. Centeno E. (2017). DisGeNET: a comprehensive platform integrating information on human disease-associated genes and variants. Nucleic Acids Res. 45, D833–D839. 10.1093/nar/gkw943 27924018 PMC5210640

[B72] Rampadarath A. Balogun F. O. Sabiu S. (2023). Insights into the mechanism of action of helianthus annuus (sunflower) seed essential oil in the management of type-2 diabetes mellitus using network pharmacology and molecular docking approaches. Endocrines 4, 327–349. 10.3390/endocrines4020026

[B73] Sandjo L. P. Poumale H. M. P. Siwe X. N. Ntede H. N. Shiono Y. Ngadjui B. T. (2011). Two new fatty acid derivatives from the stem bark of alchornea laxiflora (euphorbiaceae). JAOCS, J. Am. Oil Chem. Soc. 88, 1153–1159. 10.1007/s11746-011-1770-7

[B74] Santos M. Lima L. Carvalho S. Mota-Pereira J. Pimentel P. Maia D. (2023). The impact of BDNF, NTRK2, NGFR, CREB1, GSK3B, AKT, MAPK1, MTOR, PTEN, ARC, and SYN1 genetic polymorphisms in antidepressant treatment response phenotypes. Int. J. Mol. Sci. 24, 6758. 10.3390/ijms24076758 37047730 PMC10095078

[B75] Sekine A. (2014). in Chapter 10- Multiple Small Brain Metastases With Limited Focal Brain Edema From Non-Small Cell Lung Cancer With Epidermal Growth Factor Receptor Mutations (San Diego, CA, USA: Academic Press).

[B76] Shamsi S. Anjum H. Shahbaaz M. Khan M. S. Ataya F. S. Alamri A. (2022). A computational study on active constituents of Habb-ul-aas and Tabasheer as inhibitors of SARS-CoV-2 main protease. J. Biomol. Struct. Dyn. 40, 7702–7713. 10.1080/07391102.2021.1900920 33759703

[B77] Shamsol Azman A. N. Tan J. J. Abdullah M. N. Bahari H. Lim V. Yong Y. K. (2023). Network pharmacology and molecular docking analysis of active compounds in tualang honey against atherosclerosis. Foods 12, 1779. 10.3390/foods12091779 37174317 PMC10178747

[B78] Shannon P. Markiel A. Ozier O. Baliga N. S. Wang J. T. Ramage D. (2003). Cytoscape: a software environment for integrated models of biomolecular interaction networks. Genome Res. 13, 2498–2504. 10.1101/gr.1239303 14597658 PMC403769

[B79] Shi Y. Chen M. Zhao Z. Pan J. Huang S. (2021). Network pharmacology and molecular docking analyses of mechanisms underlying effects of the cyperi rhizoma-chuanxiong rhizoma herb pair on depression. Evid. Based. Complement. Altern. Med. 2021, 5704578. 10.1155/2021/5704578 PMC8716227 34976096

[B80] Silvestro S. Bramanti P. Mazzon E. (2021). Role of quercetin in depressive-like behaviors: findings from animal models. Appl. Sci. 11, 7116. 10.3390/app11157116

[B81] Stelzer G. Rosen N. Plaschkes I. Zimmerman S. Twik M. Fishilevich S. (2016). The GeneCards suite: from gene data mining to disease genome sequence analyses. Curr. Protoc. Bioinforma. 54, 1. 10.1002/cpbi.5 27322403

[B82] Su W.-J. Zhang Y. Chen Y. Gong H. Lian Y.-J. Peng W. (2017). NLRP3 gene knockout blocks NF-κB and MAPK signaling pathway in CUMS-induced depression mouse model. Behav. Brain Res. 322, 1–8. 10.1016/j.bbr.2017.01.018 28093255

[B83] Szklarczyk D. Gable A. L. Lyon D. Junge A. Wyder S. Huerta-Cepas J. (2019). STRING v11: protein–protein association networks with increased coverage, supporting functional discovery in genome-wide experimental datasets. Nucleic Acids Res. 47, D607–D613. 10.1093/nar/gky1131 30476243 PMC6323986

[B84] Trott O. Olson A. J. (2010). AutoDock Vina: improving the speed and accuracy of docking with a new scoring function, efficient optimization, and multithreading. J. Comput. Chem. 31, 455–461. 10.1002/jcc.21334 19499576 PMC3041641

[B85] Wal A. Wal P. Gupta D. Kumar A. Sharma P. Jain N. K. (2022). Natural products: a rising star for treating primary dysmenorrhea? Tradit. Med. Res. 7, 55. 10.53388/tmr20220410001

[B86] Wal A. Wal P. Vig H. Jain N. K. Rathore S. Krishnan K. (2023a). Treatment of Parkinson’s disease: current treatments and recent therapeutic developments. Curr. Drug Discov. Technol. 20, 10.2174/1570163820666230512100340 37183475

[B87] Wal P. Vig H. Wal A. Rathore S. Pandey S. S. Jain N. K. (2023b). Role of nutraceuticals and physical activity in Parkinson’s disease risk and lifestyle management. Curr. Aging Sci. 16, 170–187. 10.2174/1874609816666230515121717 37638586

[B88] Wang G. Li Y. Lei C. Lei X. Zhu X. Yang L. (2021a). Quercetin exerts antidepressant and cardioprotective effects in estrogen receptor α-deficient female mice via BDNF-AKT/ERK1/2 signaling. J. Steroid Biochem. Mol. Biol. 206, 105795. 10.1016/j.jsbmb.2020.105795 33246157

[B89] Wang J. Chen R. Liu C. Wu X. Zhang Y. (2021b). Antidepressant mechanism of catalpol: involvement of the PI3K/Akt/Nrf2/HO-1 signaling pathway in rat hippocampus. Eur. J. Pharmacol. 909, 174396. 10.1016/j.ejphar.2021.174396 34332921

[B90] Wang J. Q. Mao L. (2019). The ERK pathway: molecular mechanisms and treatment of depression. Mol. Neurobiol. 56, 6197–6205. 10.1007/s12035-019-1524-3 30737641 PMC6684449

[B91] Wang Y. Yang Z. Wang W. Huang Y. Zhang Q. Li J. (2022). Methodology of network pharmacology for research on Chinese herbal medicine against COVID-19: a review. J. Integr. Med. 20, 477–487. 10.1016/j.joim.2022.09.004 36182651 PMC9508683

[B92] Wang Y. Zhang Y. Wang Y. Shu X. Lu C. Shao S. (2021c). Using network pharmacology and molecular docking to explore the mechanism of Shan ci gu (cremastra appendiculata) against non-small cell lung cancer. Front. Chem. 9, 682862. 10.3389/fchem.2021.682862 34178945 PMC8220148

[B93] Wei Y. Qi K. Yu Y. Lu W. Xu W. Yang C. (2021). Analysis of differentially expressed genes in the dentate gyrus and anterior cingulate cortex in a mouse model of depression. Biomed. Res. Int. 2021, 5013565. 10.1155/2021/5013565 33628784 PMC7892236

[B94] Wu D. Shi J. Elmadhoun O. Duan Y. An H. Zhang J. (2017). Dihydrocapsaicin (DHC) enhances the hypothermia-induced neuroprotection following ischemic stroke via PI3K/Akt regulation in rat. Brain Res. 1671, 18–25. 10.1016/j.brainres.2017.06.029 28684048

[B95] Wubetu M. Sintayehu M. Abdelwuhab M. (2018). Ethnobotany of medicinal plants used to treat various mental illnesses in Ethiopia: a systematic review. Asian J. Plant Sci. Res. 14

[B96] Xia J. Gu L. Guo Y. Feng H. Chen S. Jurat J. (2021). Gut microbiota mediates the preventive effects of dietary capsaicin against depression-like behavior induced by lipopolysaccharide in mice. Front. Cell. Infect. Microbiol. 11, 627608. 10.3389/fcimb.2021.627608 33987106 PMC8110911

[B97] Xiao X. Luo F. Fu M. Jiang Y. Liu S. Liu B. (2022). Evaluating the therapeutic role of selected active compounds in Plumula Nelumbinis on pulmonary hypertension via network pharmacology and experimental analysis. Front. Pharmacol. 13, 977921. Available at:. 10.3389/fphar.2022.977921 36059960 PMC9428143

[B98] Yagci Z. B. Esvap E. Ozkara H. A. Ulgen K. O. Olmez E. O. (2019). “Chapter Five - inflammatory response and its relation to sphingolipid metabolism proteins: chaperones as potential indirect anti-inflammatory agents,” in Molecular chaperones in human disorders. Editor Donev S. B. (Academic Press), Cambridge, UK. 10.1016/bs.apcsb.2018.09.004 30635081

[B99] Yang C. Sun N. Ren Y. Sun Y. Xu Y. Li A. (2012). Association between AKT1 gene polymorphisms and depressive symptoms in the Chinese Han population with major depressive disorder. Neural Regen. Res. 7, 235–239. 10.3969/j.issn.1673-5374.2012.03.014 25767506 PMC4353122

[B100] Yang H. Wang L. Zang C. Wang Y. Shang J. Zhang Z. (2020). Src inhibition attenuates neuroinflammation and protects dopaminergic neurons in Parkinson’s disease models. Front. Neurosci. 14, 45. Available at:. 10.3389/fnins.2020.00045 32132891 PMC7040487

[B101] Ye J. Li L. Hu Z. (2021). Exploring the molecular mechanism of action of yinchen wuling powder for the treatment of hyperlipidemia, using network pharmacology, molecular docking, and molecular dynamics simulation. Biomed. Res. Int. 2021, 9965906. 10.1155/2021/9965906 34746316 PMC8568510

[B102] Ye S. Zhong J. Huang J. Zhang S. Li H. Chen D. (2020). (+)4-Cholesten-3-one promotes differentiation of neural stem cells into dopaminergic neurons through TET1 and FoxA2. Neurosci. Lett. 735, 135239. 10.1016/j.neulet.2020.135239 32650052

[B103] Ye Y. Yao S. Wang R. Fang Z. Zhong K. Nie L. (2019). PI3K/Akt/NF-κB signaling pathway regulates behaviors in adolescent female rats following with neonatal maternal deprivation and chronic mild stress. Behav. Brain Res. 362, 199–207. 10.1016/j.bbr.2019.01.008 30630016

[B104] Zhang H. Chen M. Liu Y. Dong X. Zhang C. Jiang H. (2020). Paroxetine combined with fluorouracil plays a therapeutic role in mouse models of colorectal cancer with depression through inhibiting IL-22 expression to regulate the MAPK signaling pathway. Exp. Ther. Med. 20, 240. 10.3892/etm.2020.9370 33178338 PMC7651781

[B105] Zhang T. Wei W. Chang S. Liu N. Li H. (2022a). Integrated network pharmacology and comprehensive bioinformatics identifying the mechanisms and molecular targets of yizhiqingxin formula for treatment of comorbidity with Alzheimer’s disease and depression. Front. Pharmacol. 13, 853375. 10.3389/fphar.2022.853375 35548356 PMC9081443

[B106] Zhang Z. Xu J. Ma S. Lin N. Hou M. Wei M. (2022b). Integration of network pharmacology and molecular docking technology reveals the mechanism of the therapeutic effect of xixin decoction on Alzheimer’s disease. Comb. Chem. High. Throughput Screen. 25, 1785–1804. 10.2174/1386207325666220523151119 35616676

